# A novel anti-cancer agent Icaritin suppresses hepatocellular carcinoma initiation and malignant growth through the IL-6/Jak2/Stat3 pathway

**DOI:** 10.18632/oncotarget.5578

**Published:** 2015-09-10

**Authors:** Hong Zhao, Yuming Guo, Shu Li, Ruiqin Han, Jianming Ying, Hai Zhu, Yuanyuan Wang, Li Yin, Yuqing Han, Lingzhi Sun, Zhaoyi Wang, Qingcong Lin, Xinyu Bi, Yuchen Jiao, Hongying Jia, Jianjun Zhao, Zhen Huang, Zhiyu Li, Jianguo Zhou, Wei Song, Kun Meng, Jianqiang Cai

**Affiliations:** ^1^ Department of Abdominal Surgical Oncology, Cancer Hospital, Chinese Academy of Medical Sciences and Peking Union Medical College, Beijing, P.R. China; ^2^ Beijing Shenogen Biomedical Co., Ltd, Beijing, P.R. China; ^3^ National Laboratory of Medical Molecular Biology, Institute of Basic Medical Sciences, Chinese Academy of Medical Sciences and Peking Union Medical College, Beijing, P.R. China; ^4^ Department of Pathology, Cancer Hospital, Chinese Academy of Medical Sciences and Peking Union Medical College, Beijing, P.R. China; ^5^ Laboratory of Cell and Molecular Biology & State Key Laboratory of Molecular Oncology, Cancer Institute and Cancer Hospital, Chinese Academy of Medical Sciences and Peking Union Medical College, Beijing, P.R. China

**Keywords:** Icaritin, HCC, HCC initiating cells, IL-6 receptors, Stat3

## Abstract

Tumor-initiating cell (TIC) is a subpopulation of cells in tumors that are responsible for tumor initiation and progression. Recent studies indicate that hepatocellular carcinoma-initiating cells (HCICs) confer the high malignancy, recurrence and multi-drug resistance in hepatocellular carcinoma (HCC). In this study, we found that Icaritin, a prenylflavonoid derivative from Epimedium Genus, inhibited malignant growth of HCICs. Icaritin decreased the proportion of EpCAM-positive (a HCICs marker) cells, suppressed tumorsphere formation *in vitro* and tumor formation *in vivo*. We also found that Icaritin reduced expression of Interleukin-6 Receptors (IL-6Rs), attenuated both constitutive and IL-6-induced phosphorylation of Janus-activated kinases 2 (Jak2) and Signal transducer and activator of transcription 3 (Stat3), and inhibited Stat3 downstream genes, such as Bmi-1 and Oct4. The inhibitory activity of Icaritin in HCICs was augmented by siRNA-mediated silencing of Stat3 but attenuated by constitutive activation of Stat3. Taken together, our results indicate that Icaritin is able to inhibit malignant growth of HCICs and suggest that Icaritin may be developed into a novel therapeutic agent for effective treatment of HCC.

## INTRODUCTION

Human hepatocellular carcinoma (HCC) is the fifth most common cancer type and the third leading cause of cancer death worldwide [1]. Similar incidence of HCC is observed in both developing and developed countries [2]. Currently, surgical resection and transplantation still are main approaches to treat HCC while most of HCCs are inoperable due to advanced stages. In addition, after surgical resection, the long-term prognosis of HCC is still poor and recurrence remains a major problem [3]. Chemotherapeutics, such as Cisplatin and its relatives, are also used to treat patients with advanced stage of HCC [4]. However, chemoresistance and drug toxicity in patients prevent long-term usage of these chemo drugs [4]. Therefore, alternative approaches using effective and less toxic agents are urgently required for HCC treatment.

Tumor-initiating cells (TICs) or tumor stem/progenitor cells are a subpopulation of cancer cells that have stem cell characteristics and are indispensable for tumorigenesis. TICs are most resistant to conventional cancer therapies such as chemo- and radio-therapy [5]. Accumulating evidence suggests the existence of TICs in various cancers, from leukemia to solid tumors, such as brain, colon, breast, prostate cancer and melanoma [6-10]. Recently, the expression of several molecular markers, including EpCAM, CD133 and CD24, has been identified as biomarkers for HCC initiating cells [11-13].

Signal transducer and activator of transcription 3 (Stat3) stimulates diverse cellular process triggered by several extracellular cytokines and growth factors, such as IL-6 [14]. Activated by the Janus-activated kinases (Jaks), Stat3 translocates to nucleus to induce expression of target genes such as Mcl-1, and CyclinD1 [14]. The IL-6/Stat3 pathway is involved in the development of different types of solid tumor and the growth of TICs [15, 16]. In HCC, the IL-6/Stat3 signaling pathway is involved in the maintenance and proliferation of HCICs [11, 17-19]. Recently, studies indicated that the stemness genes Bmi-1 and Oct4 are involved in HCC initiation [20, 21], and their expression was up-regulated by the IL-6/Stat3 signaling [22-24]. Therefore, agents that attenuate the IL-6/Stat3 signaling pathway would potentially inhibit growth of HCICs.

Icaritin is a prenylflavonoid derivative from Epimedium Genus that has been used in Chinese traditional medicine for long time. Previous studies were mainly focused on Icaritin's activities in the protection of neuron, the promotion of cardiac differentiation, and the prevention of steroid-associated osteonecrosis [25-27]. Recently, increasing evidence indicated that Icaritin is a novel anti-cancer agent for different kind of cancers. Icaritin inhibits the growth and enhances the radio-sensitivity of breast cancer cells, induces apoptosis of human endometria cancer cells, exhibits potent growth inhibitory activity in chronic myeloid leukemia, and suppresses renal cell carcinoma growth [28-32]. In addition, in phase I study of Icaritin in human HCC, five patients acquired partial response (PR) or stable disease (SD) among thirteen HCC patients [33]. However, the potential therapeutic activities and the underlying mechanism of Icaritin in HCC and especially in hepatocellular carcinoma initiating cells (HCICs) have not been established yet. In this study, we investigated the inhibitory function of Icaritin in the malignant growth of HCC and demonstrated that this inhibition activity functions through attenuating the IL-6Rs/Jak2/Stat3 signaling pathway.

## RESULTS

### Icaritin inhibits growth of HCC cells

**Figure 1:**
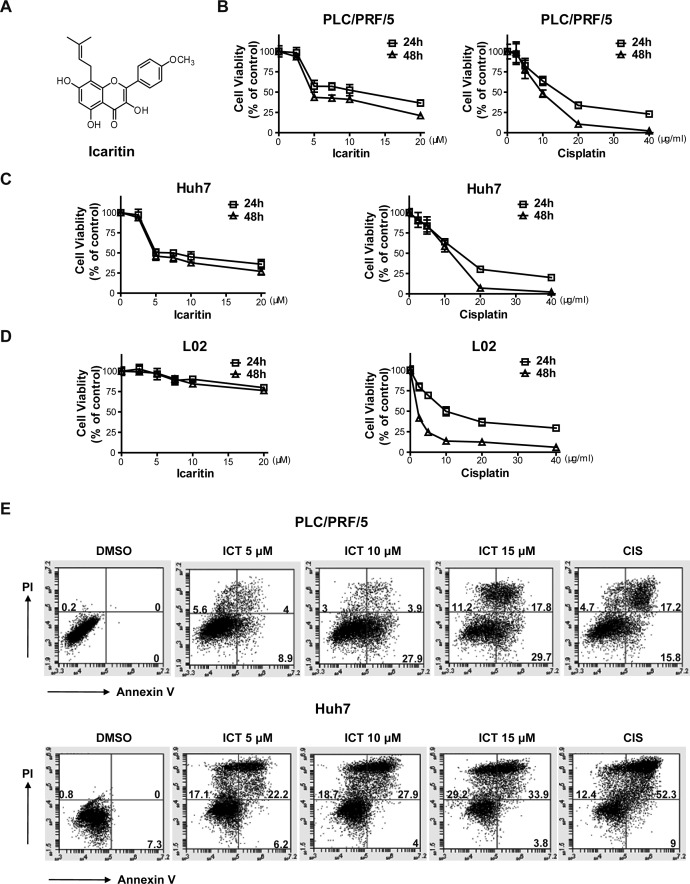
Icaritin treatment inhibits growth and induces apoptosis in HCC cells (A). Chemical structure of Icaritin. PLC/PRF/5 (B), Huh7 (C) and L02 (D) cells were treated with the indicated concentrations of Icaritin (Left) or Cisplatin (Right) for 24 and 48 h respectively and cell growth was measured by the CCK8 assay. Point, mean (n=3); bars, SD. Data is expressed as percent of vehicle (DMSO) control. (E). PLC/PRF/5 and Huh7 cells were treated with the indicated concentrations of Icaritin or Cisplatin (40 μg/ml) for 12h and then collected for apoptosis assay. The results represent two independent experiments.

Icaritin is a prenylflavonoid derivative and its chemical structure is shown (Figure 1A) [28]. We first tested the effects of different concentrations of Icaritin on growth of established HCC cell lines with the CCK8 assay. We found that Icaritin effectively inhibited PLC/PRF/5 and Huh7 cells viability in a dose- and time-dependent manner (Figure 1B, 1C Left). However, Icaritin exhibited little inhibitory activity in the L02 normal hepatocyte cell line (Figure 1D, Left). These results indicated that Icaritin inhibits the growth of HCC cells but not normal hepatocytes. However, the chemotherapy agent, Cisplatin, exhibited strong growth inhibitory activity in HCC cells as well as in L02 cells without selectivity (Figure 1B-1D, Right). To determine whether Icaritin-induced growth inhibition of HCC is through apoptosis, we performed Annexin Vapoptosis assays. We found Icaritin induced cell apoptosis with a dose-dependent manner in PLC/PRF/5 and Huh7 cells (Figure 1E, S1).

### Icaritin reduces the population of HCC initiating cells

**Figure 2:**
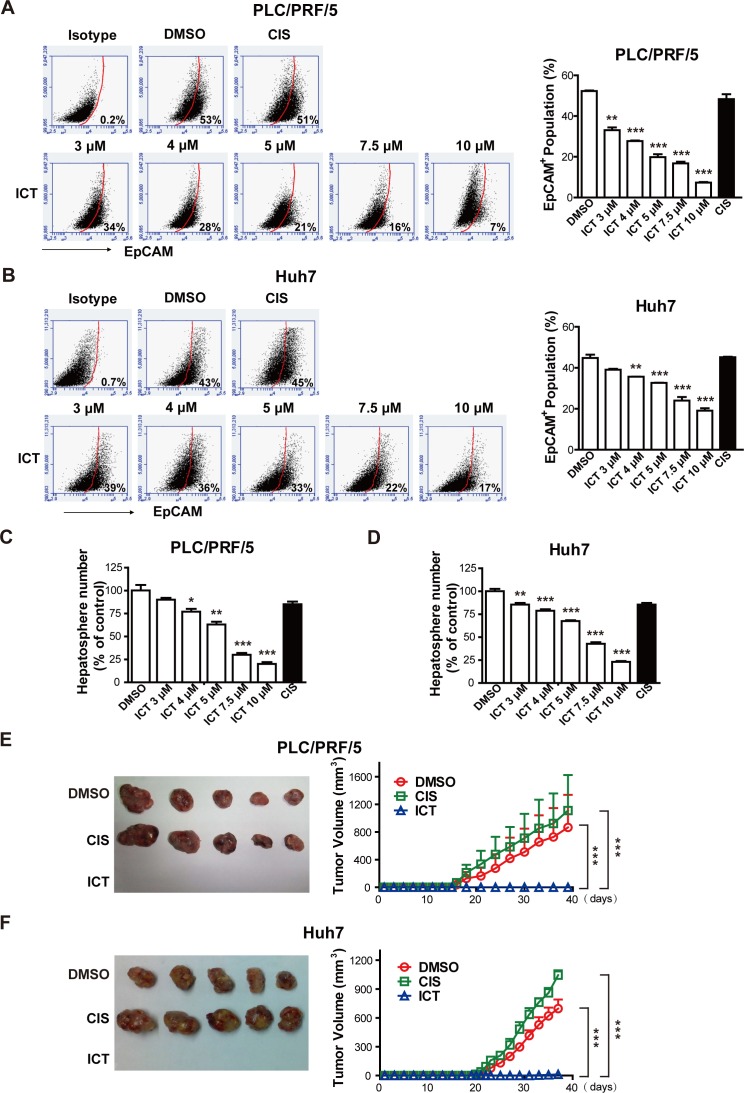
Icaritin suppresses HCICs (A, B). PLC/PRF/5 and Huh7 cells were treated with the indicated concentrations of Icaritin, Cisplatin (10μg/ml) or DMSO for two days and then the 7-AAD negative cells (living cells) were assayed with EpCAM flow cytometric analysis. (C, D). PLC/PRF/5 and Huh7 cells were treated with Icaritin, Cisplatin or DMSO for two days, washed and maintained in normal medium for another one day for recovering. The 7-AAD negative cells were seeded in low attachment dishes in the absence of drugs to form hepatospheres for five days. The number of the hepatoshperes was counted. Columns, mean (n = 3); bars, SEM. (E, F). PLC/PRF/5 and Huh7 cells were treated with Icaritin (10 μM), Cisplatin (10 μg/ml) or DMSO for two days, washed and cells were cultured for another one day for recovering, 5×10^5^ of 7-AAD negative cells were injected subcutaneously in each of NOD/SCID mice in the absence of drugs. Points, mean (n=5); bars, SEM. *P< 0.05; **P< 0.01; ***P<0.001; ICT, Icaritin; CIS, Cisplatin.

TICs have been identified and characterized using specific cell surface markers. CD34^+^ was used for leukemia-initiating cells [7], and CD44^+^CD24^−^ or ALDH1^+^ was used to enrich breast cancer-initiating cells [6, 7, 34]. EpCAM was identified as a marker for HCC initiating cells (HCICs) and EpCAM^+^ HCC cells efficiently form non-adherent spherical clusters of cells *in vitro*, termed hepatospheres and efficiently initiate tumors in NOD/SCID mice [13]. Thus, we sought to assess whether Icaritin reduces the EpCAM^+^ population in HCC cells. We found that Icaritin efficiently decreased the EpCAM^+^ proportion in both PLC/PRF/5 and Huh7 cells in a dose-dependent fashion (Figure 2A, 2B, and S2). Icaritin at 3 μM started to reduce EpCAM^+^ cells, and at 10 μM Icaritin generated over 60% reduction of EpCAM^+^ cells compared to the vehicle–treated cells (Figure 2A, 2B). Cisplatin, however, had no such effect (Figure 2A, 2B, and S2). The steady state mRNA levels of other HCICs markers such as CD133 and CD24 were also reduced by Icaritin (Figure S3).

Next, we investigated the stem-like cell properties of HCC after Icaritin treatment. First, we observed that cells from Icaritin-treated group subsequently formed less number and smaller size of hepatospheres than cells from vehicle- or Cisplatin-treated group *in vitro* (Figure 2C, 2D and S4). We then assessed the tumor-initiating ability of the cells pre-treated with Icaritin. The results showed that Icaritin pretreatment totally abolished tumor formation in mice (Figure 2E, 2F). On the contrary, the cells pre-treated with vehicle or Cisplatin still formed tumors efficiently (Figure 2E, 2F). Furthermore, serial transplant tumorigenesis assay with cells from Icaritin-treated group demonstrated Icaritin is able to reduce the population of HCICs (Table 1).

**Table 1:** Tumor seeding ability with serial transplantation from drugs-treated HCC cells

Cells injected	PLC/PRF/5			Huh7		
	DMSO	Icaritin	Cisplatin	DMSO	Icaritin	Cisplatin
5×10^5^ (n=5)	5/5	0/5	5/5	5/5	0/5	5/5
5×10^4^ (n=5)	5/5	0/5	5/5	3/5	0/5	3/5

Cells survived from drugs treatment were subcutaneously injected with 5×10^5^ and 5×10^4^ respectively into each of NOD/SCID mice. Tumor incidence was analyzed after 40 days of cells injection.

### Icaritin attenuates the Stat3 signaling pathway in HCC cells

**Figure 3:**
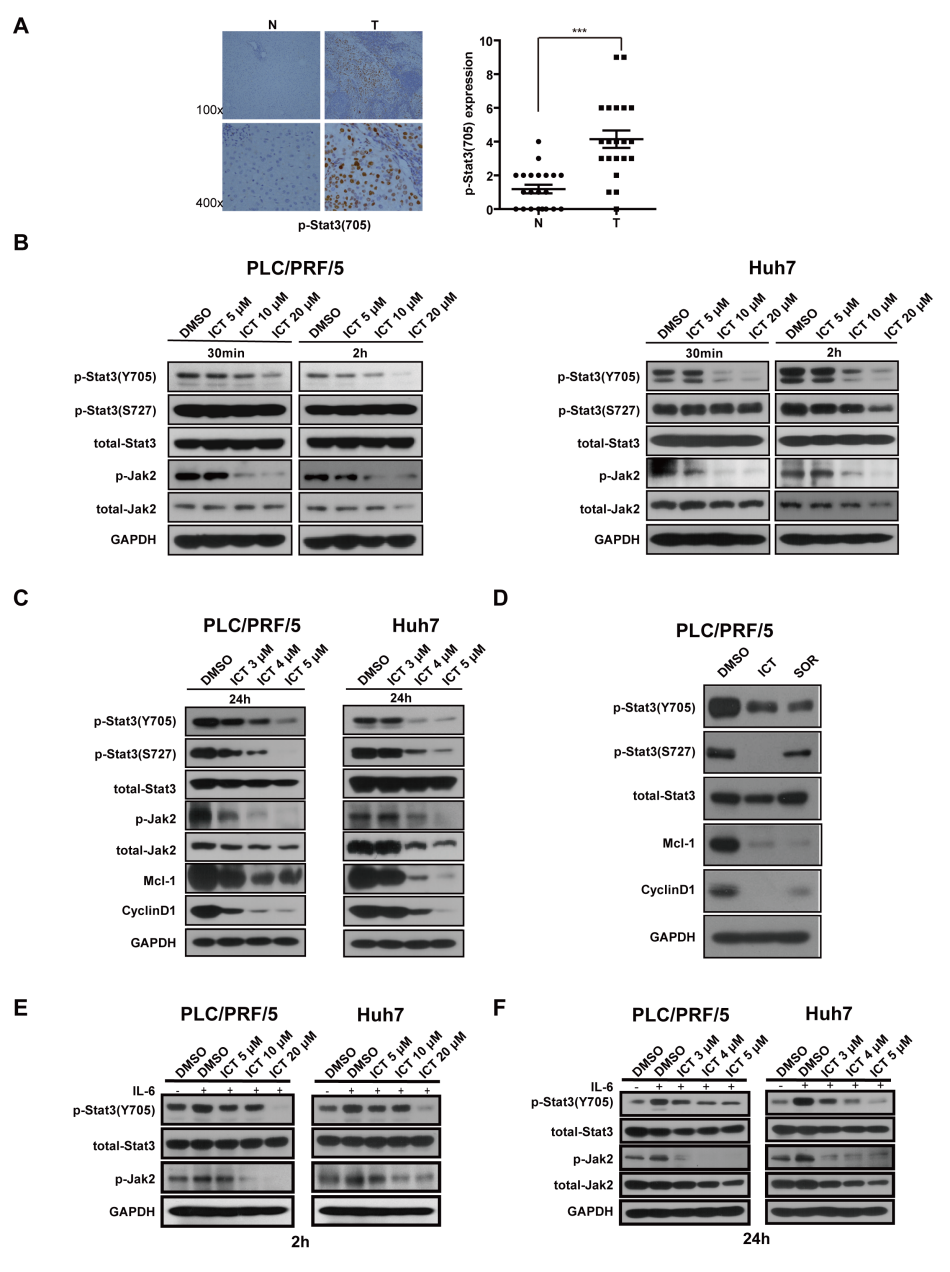
Icaritin inhibits the Stat3 signaling pathway in HCC cells (A). High level of p-Stat3 (Y705) in HCC cells. Representative images of p-Stat3 (Y705) IHC staining are shown (Left). N: adjacent non-tumor tissues, T: HCC. Magnification: 100× (upper panel); 400× (bottom panel). Statistical analysis of p-Stat3 (Y705) expression in adjacent non-tumor (N) and human HCC (T) tissues was shown. The immunoreactive score is reported as mean ± SEM (***P<0.001). (B). PLC/PRF/5 and Huh7 cells were treated with Icaritin at the indicated concentrations for 30 min or 2 h respectively. Cell lysates were prepared and western blots were performed using the indicated antibodies. (C). PLC/PRF/5 and Huh7 cells were treated with the indicated concentrations of Icaritin for 24 h. Western blots were then performed. (D). PLC/PRF/5 cells were treated with DMSO or Icaritin (5 μM) or Sorafenib (2.5 μM) for 24 h. Western blots were performed using the indicated antibodies. (E, F). Icaritin blocked IL-6-induced Stat3 phosphorylation. Cells were treated with DMSO or the indicated concentrations of Icaritin for 2 h (E) or 24 h (F) before IL-6 (1ng/ml) was added for 1 h. Western blots were then performed. ICT, Icaritin; SOR, Sorafenib.

The involvement of Stat3 signaling pathway in the maintenance of HCICs has been well documented [11, 35]. Consistence with these findings, we found that the level of phosphorylation of Stat3 at Y705 was higher in tumor tissues compared with the paired neighboring tissues (Figure 3A). Since Icaritin suppressed initiating cells of HCC, to probe the underlying mechanism, we sought to examine the effect of Icaritin on the Stat3 pathway.

We found that Icaritin attenuated p-Stat3 (Y705) phosphorylation while total Stat3 had little change (Figure 3B). Next, we performed a gene knockdown experiment using the siRNA against Jak2 and found that knockdown of the Jak2 attenuated the Stat3 phosphorylation, suggesting Jak2 stimulates Stat3 phosphorylation in HCC cells (Figure S5). Icaritin potently suppressed Jak2 phosphorylation in HCC cells. In addition, we also observed a decrease of the steady state level of Jak2 protein in Western blot analysis (Figure 3B, 3C). Furthermore, Icaritin treatment reduced the mRNA level of the Jak2, suggesting gene expression regulatory mechanism also was involved in addition to modulation of kinase activity (Figure S6).

In the cells treated with Icaritin for 2 hours, phosphorylation of the Stat3 at the residue S727 was without significant change. However, p-Stat3 (S727) was significantly reduced when cells were treated with Icaritin for 24 h (Figure 3C), suggesting that Icaritin may inhibit Stat3 phosphorylation at Ser^727^ and Tyr^705^ with different mechanisms. We also found Icaritin inhibited p-ERK1/2 in a dose-dependent manner with a similar kinetics to p-Stat3 (S727) (Figure S7A). The level of Stat3 phosphrylation at the S727 residue was attenuated in the cells treated with UO126, a MEK inhibitor (Figure S7B), suggesting Icaritin blocked ERK1/2 phosphorylation and then attenuated phosphorylation of the Stat3 at S727. The expression of the Stat3's downstream genes, Mcl-1 and CyclinD1 were also significantly reduced in the PLC/PRF/5 and Huh7 cells treated with Icaritin (Figure 3C).

Sorafenib is a chemical drug currently used for HCC treatment and it was reported that Sorafenib inhibits the activation of the Stat3 signaling [36]. Sorafenib reduced HCC cell viability dose-dependently and the IC50 of Sorafenib and Icaritin is about 2.5 μM and 5 μM, respectively (Figure S8A). Like Icaritin, Sorafenib also inhibited HCICs (Figure S8B). In Figure 3D, we show that both Sorafenib and Icaritin attenuated Stat3 phosphorylation at Y705 and reduced the expression of Stat3 downstream genes, Mcl-1 and CyclinD1. At IC50 concentrations, Icaritin reduced Stat3 (S727) phosphorylation more potently than Sorafenib in HCC. The chemotherapy agent, Cisplatin was not able to influence Stat3 phosphorylation (Figure S9).

### Icaritin inhibits IL-6-induced Stat3 phosphorylation in HCC cells

**Figure 4:**
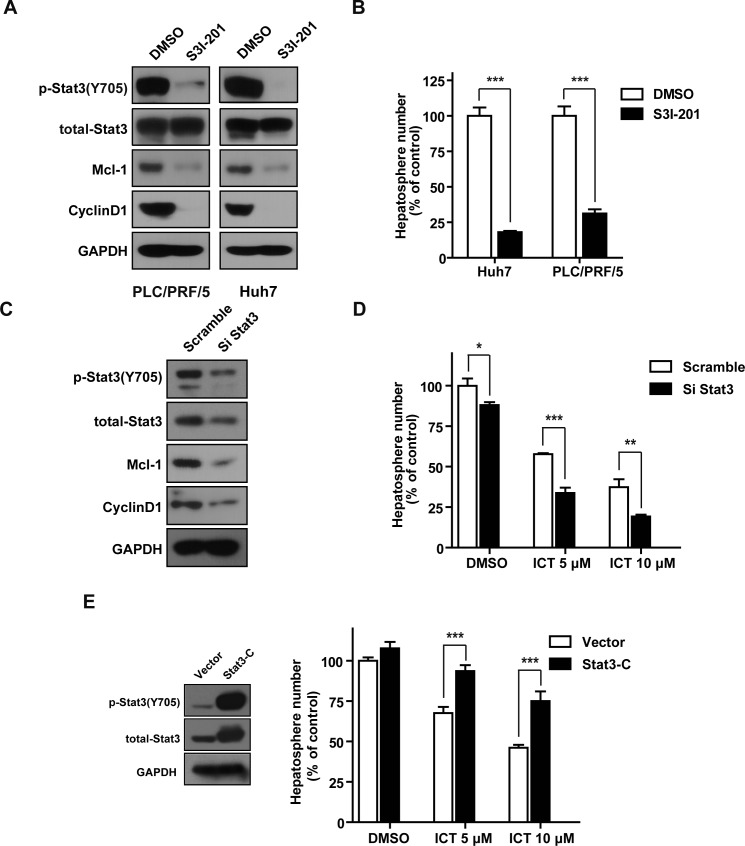
Stat3 is critical for HCC initiation and is involved in Icaritin-reduced hepatosphere formation (A). PLC/PRF/5 and Huh7 cells were treated with DMSO control or 150 μM of S3I-201 for 48 h. Western blots were performed with the indicated antibodies. (B). PLC/PRF/5 and Huh7 cells were treated with DMSO control or 150 μM of S3I-201 for 48 h. The living cells were sorted with FACS and used for the hepatosphere formation assay in the absence of S3I-201. (C). PLC/PRF/5 cells were transfected with control siRNA or the Stat3 siRNA for 48 h. Total cell lysates were prepared and analyzed with western blots using the indicated antibodies. (D). PLC/PRF/5 cells were transiently transfected with the control siRNA or the Stat3 siRNA for 48 h. The transfected cells were then used for hepatosphere formation assay in the presence of the indicated concentrations of Icaritin. Data are expressed as percent of the control. (E). PLC/PRF/5 cells were transiently transfected with the vector control or the Stat3-C expression plasmid for 48 h. Cells were harvested for western blots assay and hepatosphere formation assay. Data are expressed as percent of the vector control. Columns, mean (n = 3); bars, SEM. *, *P* < 0.05; **, *P*< 0.01; ***, *P*<0.001; ICT, Icaritin.

IL-6 is a potent cytokine that stimulates HCC progression, primarily through the Stat3 signaling [14, 37]. We observed IL-6 is highly expressed in HCC tumor tissue compared with normal liver tissue (Figure S10A). We then examined whether Icaritin is able to block the IL-6-induced Stat3 phosphorylation in HCC cells. IL-6 induced Stat3 (Y705) phosphorylation (Figure S10B), which was blocked by Icaritin treatment at higher concentrations (5, 10, 20 μM) for 2 hours (Figure 4E), or at lower concentrations (3, 4, 5 μM) for 24 hours in PLC/PRF/5 and Huh7 cells (Figure 4F). Similar results were also observed for the phosphorylation of Jak2 (Figure 4E, 4F), indicating Icaritin inhibits the IL-6-induced activation of the Jak2/Stat3 signaling.

### Stat3 plays a critical role in the maintenance of HCICs

As Icaritin potently inhibited growth of HCICs and the Jak2/Stat3 signaling, we sought to examine whether Icaritin-attenuated Jak2/Stat3 signaling is involved in HCICs inhibition by Icaritin. We treated PLC/PRF/5 and Huh7 cells with a specific Stat3 activation inhibitor, S3I-201. We found that level of Stat3 phosphorylation at the Tyr705 as well as the expression of its downstream genes, Mcl-1 and CyclinD1 were down-regulated in the cells treated with S3I-201 (Figure 4A). S3I-201 also effectively reduced HCICs and HCC growth (Figure 4B and S11). AG490, a Jak2 specific inhibitor, showed a similar inhibitory activity in the Stat3 phosphorylation, HCC cell growth and hepatosphere formation (Figure S12). Our results indicated that the Jak2/Stat3 pathway plays a critical role in HCICs.

### Icaritin suppresses HCICs through inactivation of the Stat3 signaling

To investigate whether Stat3 inactivation is involved in Icaritin-mediated hepatosphere inhibition, we changed Stat3 activity in HCC cells with the RNAi or overexpression of constitutive active Stat3. Western blot analysis showed that transfection of the siRNA against Stat3 reduced the expression levels of Stat3 and Stat3-regulated genes (Figure 4C). In addition, Stat3 silencing augmented the inhibitory activity of Icaritin in hepatosphere formation (Figure 4D). Substitution of two cysteine residues within the C-terminal loop of the SH2 domain of Stat3 generates a constitutively active Stat3 (Stat3-C) [38]. Forced expression of Stat3-C reduced Icaritin-inhibitory activity in hepatosphere formation (Figure 4E). Accordingly, these results indicated HCICs inhibition by Icaritin is Stat3 dependent.

### Icaritin downregulates IL-6 receptor and stemness genes expression

**Figure 5:**
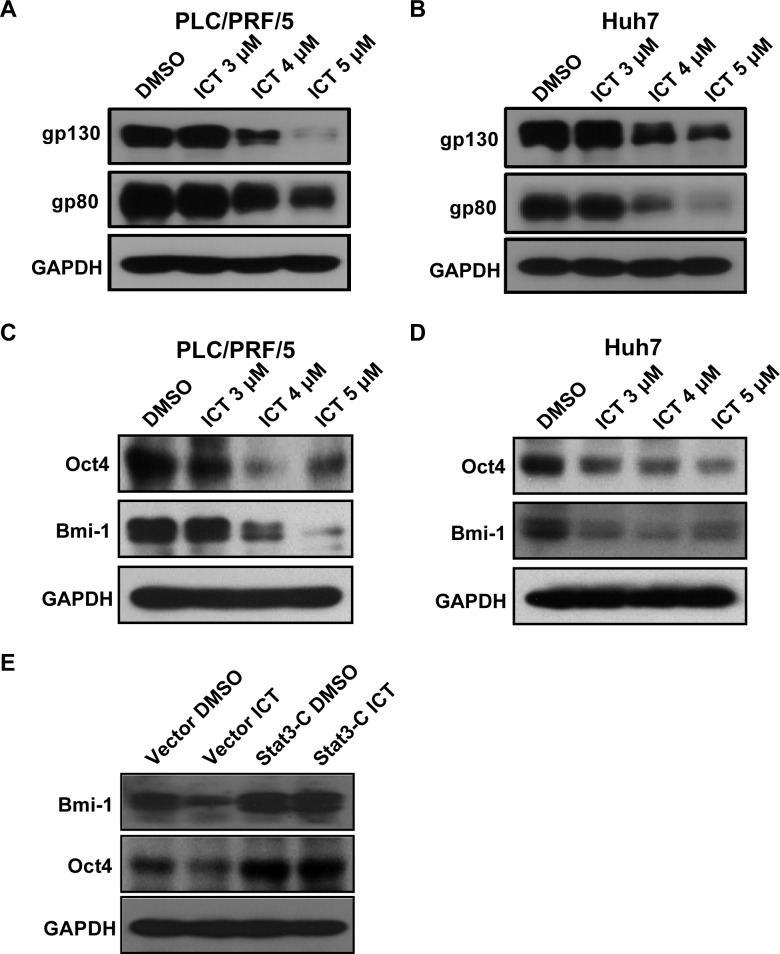
Icaritin treatment downregulates IL-6Rs expression and IL-6 signaling PLC/PRF/5 (A) and Huh7 (B) cells were treated with the indicated concentrations of Icaritin for 24 h. Total cell lysates were prepared for western blot analysis using gp130, gp80 and GAPDH antibodies. (C, D). Western blots analysis with Oct4, Bmi-1 and GAPDH antibodies. (E). Cells transfected with the vector control or the Stat3-C expression plasmid were treated with DMSO or Icaritin (4 μM) for 24 h. Western blots were performed with Bmi-1, Oct4, and GAPDH antibodies. ICT, Icaritin.

There are two types of IL-6 receptors, gp130 and gp80 (IL-6R) that mediate IL-6 –initiated signaling pathway [39]. We found that Icaritin treatment downregulated the protein levels of gp130 and gp80 in a dose-dependent manner in PLC/PRF/5 and Huh7 cells (Figure 5A, 5B), indicating that Icaritin may block the IL-6-mediated signaling pathway through downregulation of the IL-6Rs expression. IL-6Rs expression was also blocked by the Stat3 and Jak2 inhibitors, indicating that Stat3 and IL-6Rs may crossly activated each other (Figure S13).

We next studied the Icaritin's effect on the expression of HCC stemness genes. It was reported that the IL-6/Stat3 signaling pathway regulates the expression of several stemness genes, such as Bmi-1 and Oct4 [22-24]. Icaritin treatment decreased Bmi-1 and Oct4 expression in a dose-dependent manner in HCC cells (Figure 5C, 5D). In addition, the IL-6-stimulated expression of Bmi-1 and Oct4 was also attenuated by Icaritin (Figure S14). To determine whether down regulation of the Bmi-1 and Oct4 expression by Icaritin is Stat3 dependent, the constitutively active mutant Stat3-C was introduced into PLC/PRF/5 cells and the transfected cells were treated with Icaritin. Icaritin failed to inhibit Bmi-1 and Oct4 expression in the Stat3-C-transfected cells compared to the vector control cells (Figure 5E).

### Icaritin suppresses tumor formation and Stat3 phosphorylation *in vivo*

**Figure 6:**
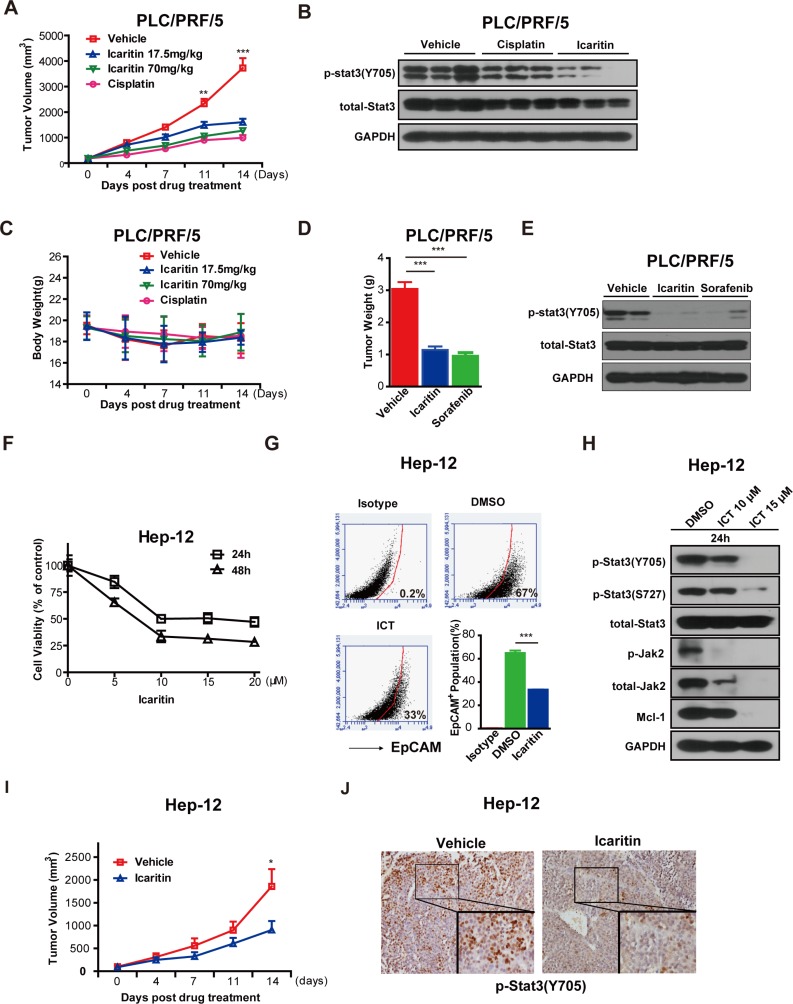
Icaritin inhibits tumor formation in xenografts of PLC/PRF/5 cells and primary HCC cells (A). NOD/SCID mice were implanted subcutaneously with 1×10^6^ PLC/PRF/5 cells per mouse. After tumors reached the volume of 100 mm^3^, the indicated concentrations of Icaritin and the vehicle control (corn oil) were administered by gastric gavage, while Cisplatin (2 mg/kg) was administered intravenously. Tumor volume (A) and mice weights (C) were measured twice a week for 14 days after drugs administration. Points, mean (n=10); bars, SEM. **P < 0.01; ***P<0.001; Vehicle group versus other groups. (B). The tumors formed by PLC/PRF/5 cells were excised and lysed. Representative results from Western blots with the antibodies of p-Stat3 (Y705), total Stat3 and GAPDH are shown. (D). After tumors formed by PLC/PRF/5 cells reached 100mm^3,^ Icaritin (70mg/kg) was administered by gastric gavage daily and Sorafenib (20mg/kg) was administrated intravenously daily. Tumor weights of each group were calculated 14 days after drug treatment. Columns, mean (n=5); bars, SEM. ***P<0.001. (E). Representative western blots results with the antibodies of p-Stat3 (Y705), total Stat3 and GAPDH. (F). Hep-12 cells were treated with DMSO (0) and the indicated concentrations of Icaritin for 24 h and48 h respectively and analyzed with the CCK8 assay. Each point represents mean (n=3) ± SD and data are expressed as percent of the DMSO control cells. (G). Hep-12 cells were treated with the DMSO control and Icaritin (7.5 μM) for 2 days and the 7-AAD negative cells were analyzed with EpCAM flow cytometry. The results represent two independent experiments. (H). Hep-12 cells were treated with the indicated concentrations of Icaritin for 24 h. Total cell lysates were prepared and western blots were performed using the indicated antibodies. GAPDH was used to ensure equal loading. (I). Effects of Icaritin on Hep-12 cells xenografts. NOD/SCID mice were implanted subcutaneously with Hep-12 cells (1×10^6^/each). Icaritin (17.5 mg/kg) or the vehicle control was administered by gastric gavage daily. Tumor volume was measured twice per week for 14 days. Points, mean (n=6); bars, SEM. *, P < 0.05; Icaritin group versus vehicle group. (J). Icaritin reduces phosphorylation of Stat3 at the Tyr705 determined by IHC staining. ICT, Icaritin.

To test Icaritin growth inhibitory activity *in vivo*, we examined the effects of Icaritin on established HCC xenografts. Icaritin administration potently inhibited the growth of the tumor formed by PLC/PRF/5 cells in NOD/SCID mice (Figure 6A), which was accompanied by a reduction of p-Stat3 (Y705) level (Figure 6B). Body weight loss was not observed in Icaritin-administrated groups, indicating Icaritin's safety (Figure 6C). Additionally, we examined the tumor incidence of secondary xenografts in NOD/SCID mice that were inoculated with equal number of cells obtained from primary vehicle-treated and Icaritin-treated xenografts. Icaritin-treated group exhibited reduced tumor occurrence (Figure S15). We also studied Sorafenib's effect in PLC/PRF/5 xenografts model. Sorafenib (20mg/kg) has the comparative effects as Icaritin (70mg/kg) in PLC/PRF/5 xenografts inhibition and down-regulation of p-Stat3 (Y705) (Figure 6D, 6E).

### Icaritin exhibits inhibitory activity in patient derived HCC cells

We then examined Icaritin's effect on growth of patient derived HCC cells. Hep-12 cells (a kind gift from Professor Baocai Xing of Cancer hospital, Peking University) were primarily cultured from a recurrent HCC patient and enriched for HCICs [40]. We found that Icaritin treatment inhibited the growth of Hep-12 cells in a dose-dependent manner *in vitro* (Figure 6F). Icaritin reduced the EpCAM positive population and the phosphorylation level of Jak2 and Stat3 in Hep-12 cells (Figure 6G, 6H). *In vivo*, Icaritin also reduced growth of Hep-12 xenografts in NOD/SCID mice (Figure 6I), which was accompanied by a down-regulation of p-Stat3 (Y705) (Figure 6J). In addition, Icaritin showed potent inhibition activity in the other two patient derived cells (Figure S16A). Icaritin reduced EpCAM^+^ population in the xenografts derived from one patient (Figure S16B).

### Icaritin inhibits hepatosphere growth through the inactivation of IL-6Rs/Jak2/Stat3 signaling

**Figure 7:**
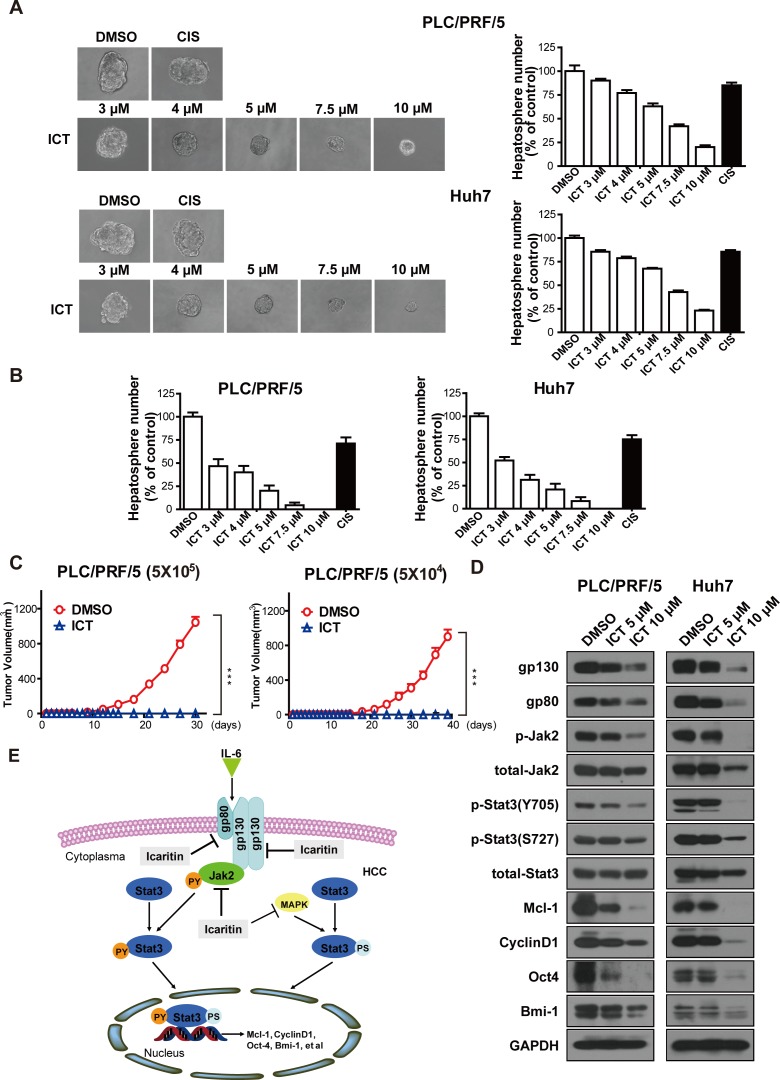
Icaritin attenuates hepatosphere growth and the IL-6/Stat3 signaling Icaritin treatment suppresses primary (A) and secondary hepatosphere (B) formation. PLC/PRF/5 and Huh7 cells were cultured in low attachment dishes. Primary hepatospheres were incubated with DMSO, 10 μg/ml Cisplatin or different concentrations of Icaritin for five days. Icaritin-treated primary hepatospheres were then dissociated to form secondary hepatospheres. Columns, mean (n = 3); bars, SEM. (C). Icaritin-treated hepatosphere cells lost tumor-initiating capability *in vivo*. Hepatospheres treated with Icaritin (10 μM) or DMSO for five days were trypsinized and injected in NOD/SCID mice subcutaneously (Left, 5×10^5^ groups; Right, 5×10^4^ groups). Points, mean (n=5); bars, SEM. ***P<0.001. (D). Icaritin suppressed the IL-6Rs/Jak2/Stat3 axis in hepatospheres. Hepatospheres of PLC/PRF/5 and Huh7 cells were treated with the indicated concentrations of Icaritin for five days and western blots assay was performed with the indicated antibodies. (E). A schematic model for Icaritin's role in HCC. IL-6 in HCC microenvironment binds to cell surface IL-6 receptors (gp80 and gp130) and activates Jak2. Icaritin blocks Jak2 phosphorylation, which mediates Stat3 tyrosine phosphorylation and downstream signaling. IL-6Rs receptors downregulated by Icaritin intensifies the attenuation of p-Stat3 (Y705). In addition, Icaritin may also suppress the MAPK/ERK signaling, that phosphorylates p-Stat3 (S727). ICT, Icaritin; CIS, Cisplatin.

HCICs are enriched in hepatosphere formation medium as non-adherent spherical clusters termed hepatospheres [11, 13]. Thus, we further studied Icaritin's effect on HCICs using the hepatosphere model. Icaritin treatment reduced both the number and size of hepatospheres formed by PLC/PRF/5 cells (Figure 7A, up). Similar results were also observed in the hepatospheres formed by Huh7 and Hep-12 cells (Figure 7A, low and S17). In addition, Icaritin attenuated the ability of hepatosphere cells to form secondary passage of hepatospheres, indicating Icaritin negatively regulates self-renewal of HCICs (Figure 7B). Furthermore, Icaritin-treated hepatospheres lost tumor initiating capability and the IL-6Rs/Jak2/Stat3 axis of hepatospheres treated with Icaritin was also suppressed (Figure 7C, 7D).

## DISCUSSION

Icaritin, a prenylflavonoid derivative from Epimedium Genus, has been used as Chinese traditional medicine for long time. Previous studies reported that Icaritin is able to reduce phosphorylation of Stat3 (Y705) in chronic myeloid leukemia and multiple myeloma [32, 41]. However, the effects of Icaritin on HCICs and Stat3's role in Icaritin-mediated HCICs inhibition have not been investigated yet. Furthermore, our study demonstrated for the first time that Icaritin targeted IL-6Rs, attenuated both Stat3 (Y705) and Stat3 (S727) and decreased the expression of IL-6-regulated stemness genes. It was reported that Icaritin activated AHR in prostate cancer [42]. Another study reported that IL-6, Stat3 and AHR may form an autocrine loop [43]. Whether AHR's activation by Icaritin mediates IL-6/Stat3 signal inhibition will need to be investigated in future.

TICs drive tumor growth and recurrence. These cells exhibit certain features *in vitro*, including expression of stem cell markers, tumorsphere formation in suspension cultures, and resistance to chemo-and radio-treatments [5]. TICs are also responsible for tumor initiation, progression, metastasis and recurrence *in vivo* [13]. Our previous study showed that Icaritin potently inhibits growth of ALDH1-positive breast tumor initiating cells [28]. Here, we showed that Icaritin abolished primary and secondary hepatosphere formation of HCICs, reduced the populations of cells positive for HCC-stem cell markers such as EpCAM, inhibited primary and secondary xenografts in NOD/SCID mice, suppressed malignant growth of the primary HCC cells, Hep-12 *in vitro* and *in vivo*. These results provide the evidence to support the view that Icaritin can be developed into a potent agent to treat HCC by targeting HCICs.

IL-6 is a cofactor important in the amplification and differentiation of stem cells [35, 44]. HCC expresses two types of IL-6 receptors, gp80 and gp130 [39]. IL-6 binds to gp80 first and then recruits gp130 to signal downstream cascades. After gp130 dimerization, Jak2 becomes activated and leads to tyrosine phosphorylation of cytoplastic transcription factors such as Stats [39]. The major Stat transcription factor activated by the IL-6 signaling is Stat3. After activation, Stat3 translocates from the cytoplasm to the nucleus and induces downstream genes. The aberrant IL-6 signaling dysregulates progenitor/stem cells, which eventually results in HCC carcinogenesis [37]. It was reported that the activated IL-6/Stat3 signaling leads upregulation of stemness genes, including Bmi-1 and Oct4, which preserve stemness of HCC cells [20-24]. In this study, using the Stat3 specific inhibitor S3I-201 or Stat3's siRNA method, we found Stat3 function is critical in tumor initiation of HCC. The HCICs inhibitory effect of Icaritin was through inactivation the Stat3 function as evidenced by the Stat3 knock-down and overexpression experiments. Our results that Icaritin blocked IL-6-induced expression of the stemness genes, Bmi-1 and Oct4 further indicated that Icaritin inhibited the IL-6/Stat3 signaling and downstream genes.

The importance of the IL-6/Stat3 signaling in cancer and cancer stem cells has been well documented. The IL-6/Stat3 signaling promotes HCC progression through inhibiting apoptosis by inducing the expression of anti-apoptotic factors of Bcl-2 family such as Mcl-1 and Bcl-xl [45]. In HCICs, Stat3 acts as a self-renewal factor in maintenance of HCICs through induction of the Oct4/Nanog stemness genes [11, 22]. Hyperactivation of the IL-6 signaling induces non-TICs convert to TICs [46]. HCICs, but not bulk HCC cells acquire the autocrine IL-6/Stat3 signaling that stimulates their malignance progression and induces HCC initiation *in vivo* [35].

Sorafenib that targets multiple kinases was approved by FDA for the advanced HCC therapy several years ago. However, the overall survival was 6.5 months in Sorafenib group and 4.2 months in the placebo group in the Asia trial [47]. Thus, less toxic and more effective agents are urgently needed for the treatment of advanced HCC. Targeted therapy with the inhibitors on several pathways, such as VEGFR, EGFR, mTOR and c-MET is in development now [48]. The combination therapy of these agents, will be a strategy for HCC treatment in future.

In this study, we discovered that Icaritin potently inhibited growth of HCC cells but has little toxicity in normal hepatocyte cells compared to Cisplatin. Icaritin (17.5mg/kg and 70mg/kg) exhibits low toxicity since animal weights were without change during the whole experiments, consistent with a previous study that Icaritin has a favorable pharmacokinetics and safety profiles [42]. Furthermore, our clinical studies [NCT01278810, NCT01972672] showed Icaritin exhibits high level of safety even after orally received 1600 mg per day. These results indicate Icaritin is a less toxic and high effective agent for HCC therapy.

Currently, a clinical phase I study with Icaritin has been completed [33]. Among thirteen HCC patients who were treated with Icaritin and evaluated, one patient obtained partial response (PR) and progressed after one-year treatment, and four patients had stable disease (SD) for more than 4 months [33]. Now, the phase II clinical study of Icaritin in HCC [NCT01972672] is currently underway. Since the therapeutic strategies for HCC are limited, our study provides a strong rational for development of Icaritin as a novel therapeutic agent for effective and safe treatment of HCC by targeting HCICs.

## MATERIALS AND METHODS

### Human tissue specimens

A total of twenty-one pairs of patient samples were used in the study. All patients received curative resection for liver cancer at Cancer Hospital, Chinese Academy of Medical Science & Peking Union Medical College (Beijing, China) between March 2014 and August 2014. The patients did not receive any preoperative cancer treatments. The clinicopathological characteristics of the patients are presented in Table S1. Clinical samples from patients were collected for immunochemistry staining after obtaining informed consent in accordance with a protocol approved by the Ethics Committee of Cancer Hospital, Chinese Academy of Medical Science & Peking Union Medical College (Beijing, China).

### Animal models

All experimental procedures were approved by The Animal Care and Use Committee of Cancer Hospital, Chinese Academy of Medical Science & Peking Union Medical College (Beijing, China). Female, 4-6 weeks old NOD/SCID mice were used (Vitalriver, Beijing, China) in animal experiments.

To perform the tumor seeding ability assay, the survived cells from HCC cells treated with DMSO, Icaritin (10μM), and Cisplatin (10 μg/mL) for 48 h or hepatospheres treated with DMSO and Icaritin (10μM) for five days were selected with flow cytometry after 7-AAD staining. Serial transplant tumorigenesis assay was performed by subcutaneously injected with 5×10^5^ or 5×10^4^ selected cells into each of NOD/SCID mice. Tumor incidence and tumor growth curves were examined after 30 days of implantation.

For *in vivo* assay, NOD/SCID mice were implanted subcutaneously with 1×10^6^ PLC/PRF/5 or Hep-12 cells. For the PDX model, xenografts maintained in nude mice within 10 passages were implanted subcutaneously. After tumors reached about 100 mm^3^, the indicated concentrations of Icaritin or vehicle control (corn oil) were administered by gastric gavage daily. Cisplatin (2 mg/kg) was administered intravenously twice per week. Sorafenib (20mg/kg) was administrated intravenously daily. Tumor growth was monitored twice per week with digital caliper measurements and tumor volume was calculated as L×W^2^/2.

For the secondary xenograft, PLC/PRF/5 primary xenografts treated with the vehicle control or Icaritin (70mg/kg) were collected and minced into 1 mm^3^ cubes and incubated with Type IV Collagenase (Sigma Aldirich) for 30 min at 37°C. A single-cell suspension was obtained by filtering through a 100 μM cell strainer (BD Biosciences).1×10^4^ living cells sorted with FACS from each xenograft were injected subcutaneously for secondary xenografts formation in the absence of Icaritin. Tumor occurrence was evaluated as relapse percentage.

### Cell lines and cell culture

The human HCC cell lines PLC/PRF/5 and Huh-7 were originally obtained from Japanese Cancer Research Bank, Tokyo, Japan and human hepatic cell line L02 was from Type Culture Collection of Chinese Academy of Sciences, Shanghai, China. Hep-12 cells were a kind gift from Dr. Baocai Xing at Cancer hospital, Peking University and were used within 10 passages [40]. Cells were cultured in complete high glucose DMEM medium (Gibco) supplemented with 10% heat-inactivated fetal bovine serum, 100 mg/ml penicillin G, and 50 μg/ml streptomycin at 37°C in a humidified atmosphere containing 5% CO2. HCC cells used in this study produce IL-6 (Figure S18).

For the drugs or chemicals-treatment, 24 h before the experiments, cells were seeded into the conditional medium (2.5% charcoal-stripped FBS, phenol-red free Gibco DMEM medium including 100 mg/ml penicillin G, and 50 μg/ml streptomycin). Cells were then treated with the indicated concentrations of drugs or chemicals for the indicated time and the final DMSO concentration in the incubation system was no more than 0.1%.

### Plasmids, siRNAs and reagents

The siRNAs for Stat3, Jak2 (pools of three specific 19-25 nt siRNAs) and control siRNA were purchased from Santa Cruz. The Constitutive active Stat3-C plasmid was a kind gift from Professor Zhijie Chang (Tsinghua University) [49]. Icaritin was purified from Epimedium at the Beijing Shenogen pharma group with over 99 % purity. Cisplatin and AG490 were obtained from Sigma Aldrich. Sorafenib was purchased from Selleck. U0126 was purchased from Cell Signaling Technology and Hoechst was from BD Pharmingen. Human recombinant IL-6 was from Peprotech and S3I-201 was purchased from Santa Cruz Biotechnology.

### Cell viability assay

Cells at the logarithmic phase were collected and 4000 cells were seeded into each well of 96-well plates and cultured for 24 h in the conditional medium described above. The indicated concentrations of drugs were added for 24 or 48 h. Finally, cell viability was measured using the CCK-8 assay according to the manufacture instructions (Dojindo). The percentage of viable cells was calculated using the following formula: cell viability (%) = (OD of treated cells/OD of control cells) ×100.

For the viability assay with Patient derived cells (Bioduro, China), cells were acquired by primary culture and expanded with DMEM medium containing 10% fetal bovine serum for less than ten passages, then finally used for cell viability assay as mentioned above.

### Cell apoptosis assay

The cells (2×10^5^/well) in six-well plates were treated with the indicated concentrations of agents for 12 h, and then were collected and washed twice in ice-cold PBS. Cell apoptosis assay was conducted using an Annexin V-FITC kit (BD pharmingen) according to the manufacture's instruction and BD Accuri™ C6 flow cytometer (Becton-Dickinson, CA, U.S.). The results were analyzed with CFlow Plus software (Becton-Dickinson, CA, U.S.).

### EpCAM flow cytometric analysis

The phycoerythrin (PE)-conjugated EpCAM (eBioscience), PE-conjugated isotype control (eBioscience) and 7-AAD (BD pharmingen) were used for EpCAM flow cytometric assay. Cells treated with the indicated concentrations of agents or DMSO control were collected and incubated in phosphate-buffered saline (PBS) containing antibodies of EpCAM, isotype, or 7-AAD for 30 min at 4°C. Cells were washed twice with cold PBS. The results were examined with BD Accuri™ C6 flow cytometer (Becton-Dickinson, CA, U.S.) and analyzed with CFlow Plus software (Becton-Dickinson, CA, U.S.).

### Hepatosphere formation assay

A total of 1000 single HCC cells were plated in Ultra Low Attachment 24-well plates (Corning). Cells were grown in DMEM/F12 medium (Invitrogen) supplemented with 4 μg/ml insulin (Sigma), B27 (Invitrogen), 20 ng/ml EGF and 20 ng/ml basic FGF (Peprotech) for five days. To obtain secondary passage of primary spheres, the primary spheres were collected, dissociated with trypsin, and re-suspended in the medium described above. The number of hepatoshpheres was counted under a Nikon Eclipse TE2000-S microscope and presented as relative percentage of the DMSO control.

### Quantitative real-time PCR (qRT-PCR)

Total RNA was isolated using Trizol reagent according to the manufacturer's protocol (Invitrogen, Carlsbad, CA). Reverse transcription reactions were performed using the AMV First Strand cDNA Synthesis Kit (NEB, USA). Real-time PCR assays were performed using an Applied Biosystems 7300 Detection System (Applied Biosystems®, CA). The real-time PCR reaction was performed according to the protocol of the Power SYBR Green PCR Master Mix (Applied Biosystems, CA). The amplification procedure: 95°C for 15 seconds, and 60°C for 60 seconds for 40 cycles. Data was analyzed using the Sequence Detection Software, Version 1.2.3 (Applied Biosystems). The amount of target cDNA was calculated and normalized with the GAPDH levels in the samples. The primers utilized for real-time PCR analysis were listed in Table S2.

### Western blots assay

Total protein was collected from the cells after various treatments. For Western blots, a previously described procedure was applied [28]. The following primary antibodies were used: p-Jak2 (Y1007/1008), total Jak2, p-Stat3 (Y705), p-Stat3 (S727), and total Stat3 were purchased from Cell Signaling Technology. Antibodies for gp130, gp80, Bmi-1, Oct4 and GAPDH were from Santa Cruz. Mcl-1 and CyclinD1 antibodies were obtained from Abcam. The secondary anti-mouse and anti-rabbit antibodies were purchased from Santa Cruz. All the experiments were performed at least twice and quantification data with statistical analysis was summarized in Figure S19.

### Plasmids and siRNAs transfection

The cells (2×10^5^/well) in a six-well plate were transfected with the indicated plasmids (2.5 μg/ml) or siRNAs (0.45 μg/ml) by Lipofectamine 3000™ following the manufacturer's instructions (Invitrogen). After 48 h, cells were harvested for the western blots, cell viability assay or hepatosphere formation assay as described above.

### Immunohistochemistry (IHC) staining

All tissues were fixed in 4% paraformaldehyde overnight at 4°C, processed, sectioned to thickness of 5 μM, and de-waxed. Antigen retrieval by heating the slides in the rice cooker for three minutes using citrate buffer (pH 6.0) was followed by treatment with 3% H_2_O_2_ for 10 minutes. The slides were then incubated with 1% BSA/PBS for 1 h at room temperature to reduce nonspecific background staining. The slides were incubated with primary antibodies overnight at 4°C, then rinsed and incubated for 1 h at room temperature with peroxidase-conjugated secondary antibody (Ventana). The slides were rinsed with PBS, incubated for 90 seconds with diaminobenzidine k8000 kit (DAKO), counterstained with hematoxylin (DAKO) and mounted in dimethyl benzene. For the p-Stat3 (Y705) scoring, cells in five fields were randomly selected and scored by two pathologists. Depending on the intensity of staining, the staining was classified into 4 groups: (no staining=0; weak staining = 1; moderate staining = 2; and strong staining = 3). The staining was also classified into 5 groups according the percentage of stained cells (0% = 0; 1%-25% = 1; 26%-50% = 2; 51%-75% =3; and 76%-100% = 4). Final immunoreactive scores were determined by the formula: overall scores = intensity score × percentage score. The overall score ≤ 3 was defined as negative, >3 as positive.

### Enzyme-linked immunosorbent (ELISA) assay for IL-6

IL-6 levels were measured using the supernatants from HCC cell condition medium or fresh medium as a control. The experiments were performed with a commercially available human IL-6 High Sensitivity ELISA kit (sensitivity 0.03 pg/ml) according to the manufacture's instruction.

### Statistic analyses

Statistical analyses were performed using the program developed by the Prism GraphPad software, Inc. (La Jolla, CA, USA). The values are represented as mean ± SD or SEM as indicated. The difference between groups was analyzed using the Student's *t*-test when comparing only two groups or by a one-way ANOVA analysis when comparing more than two groups. P<0.05 was considered statistically significant.

## SUPPLEMENTARY MATERIAL FIGURES AND TABLES



## Abbreviations

TICs: Tumor-initiating cells
HCICs: hepatocellular carcinoma-initiating cells
HCC: hepatocellular carcinoma
IL-6: Interleukin-6
IL-6Rs: Interleukin-6 Receptors
EpCAM: epithelial cell adhesion molecule
Stat3: Signal transducer and activator of transcription 3
Jaks: Janus-activated kinases
Jak2: Janus-activated kinases 2
PDX: patient derived xenograft
ERK: extracellular signal-regulated kinase
PR: partial response
SD: stable disease
qRT-PCR: quantitative real-time polymerase chain reaction
IHC: immunohistochemistry
