# Supplementary material for: A novel anti-cancer agent Icaritin suppresses hepatocellular carcinoma initiation and malignant growth through the IL-6/Jak2/Stat3 pathway
Source: Oncotarget. 2015 Sep 10;6(31):31927–43. doi: 10.18632/oncotarget.5578 (PMC4741651; doi:10.18632/oncotarget.5578)
Supplement: Supplementary file 1 [file oncotarget-06-31927-s001.pdf]

A novel anti-cancer agent Icaritin suppresses hepatocellular carcinoma initiation and malignant growth through the IL-6/Jak2/Stat3 pathway

Supplementary Material

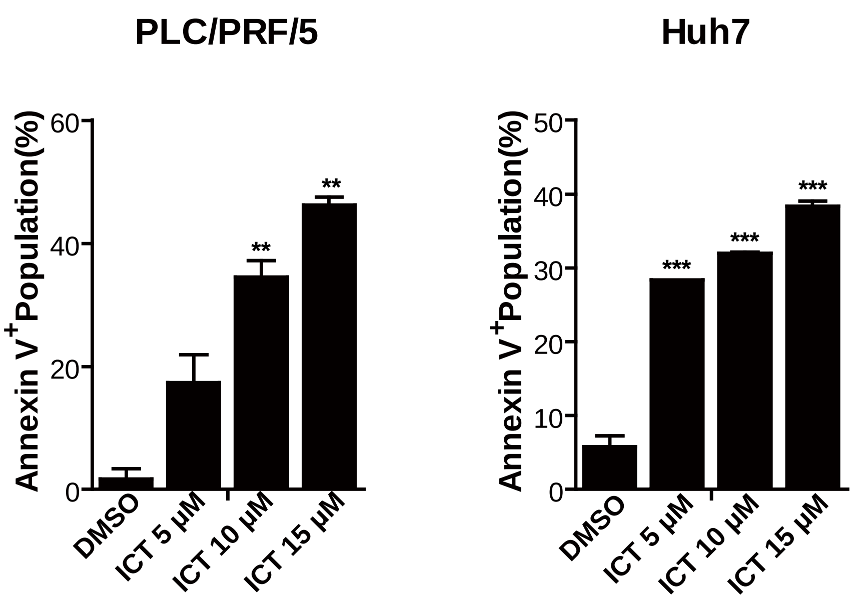

Supplementary Figure S1: Icaritin induces HCC cells apoptosis. PLC/PRF/5 and Huh7 cells were treated with Icaritin for 12 h, and then were collected for flow cytometric analysis using Annexin Ⅴ/PI assay. The population of Annexin Ⅴ positive cells was analyzed. Columns, mean (n=3); bars, SEM; **P <0.01; ****P* <0.001. ICT, Icaritin.

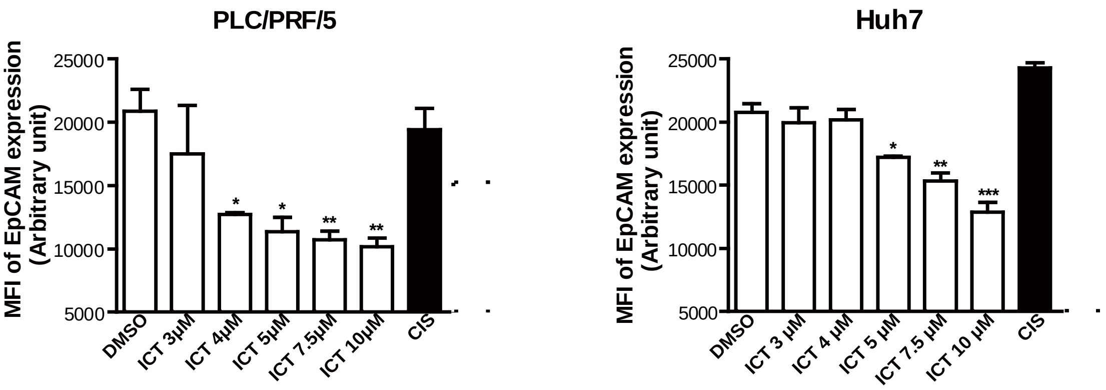

Supplementary Figure S2: Icaritin treatment decreases EpCAM mean fluorescence intensity (MFI) of HCC cells. PLC/PRF/5 and Huh7 cells were treated with the indicated concentration of Icaritin or Cisplatin (10 μg/ml) for 48 h, and then the 7-AAD negative cells were subjected to EpCAM flow cytometric analysis. Data are presented as mean fluorescence intensity (MFI) of EpCAM in HCC cells. Columns, mean (n=3); bars, SEM; **P* < 0.05; ***P* <0.01; ****P* <0.001. ICT, Icaritin; CIS, Cisplatin.

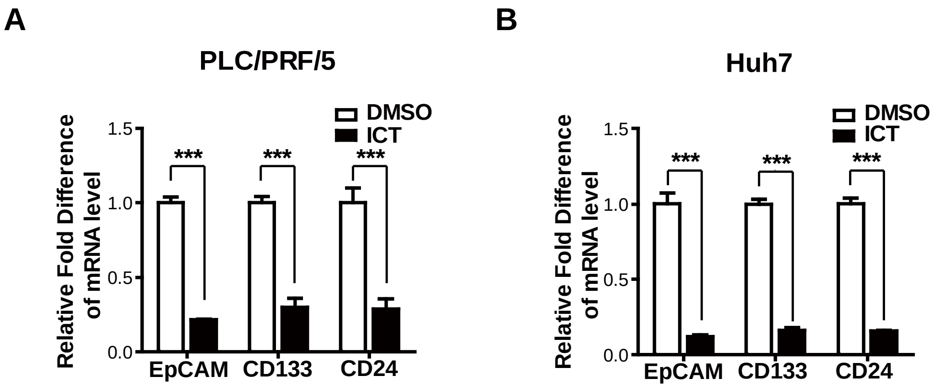

Supplementary Figure S3: Icaritin decreases the mRNA levels of EpCAM, CD133 and CD24 in PLC/PRF/5 and Huh7 cells. PLC/PRF/5 and Huh7 cells were treated with 5 μM Icaritin for 24 h then cells were subjected to qRT-PCR assay using EpCAM, CD133 and CD24 primers. Columns, mean (n = 3); bars, SEM. ***, P < 0.001; ICT, Icaritin.

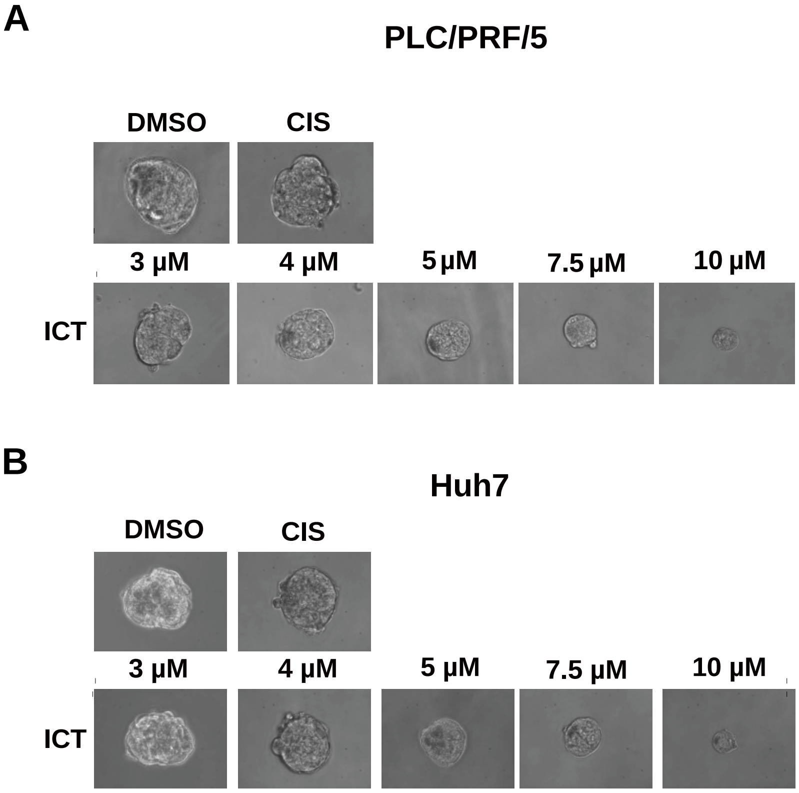

Supplementary Figure S4: Pretreatment of HCC cells with Icaritin reduces the hepatospheres size. PLC/PRF/5 (A) and Huh7 (B) cells were treated with Icaritin (3, 4, 5, 7.5, 10 μM), Cisplatin (10 μg/ml) or DMSO for 2 days in phenol red-free DMEM medium with 2.5% charcoal-stripped fetal calf serum, then drugs were removed and cells were maintained in complete DMEM medium for another 1 day for recovering, After 7-AAD positive cells excluded, cells were sorted into the sphere-forming medium in the absence of drugs and the hepatosphere size was evaluated with the phase contract microscope. ICT, Icaritin; CIS, Cisplatin.

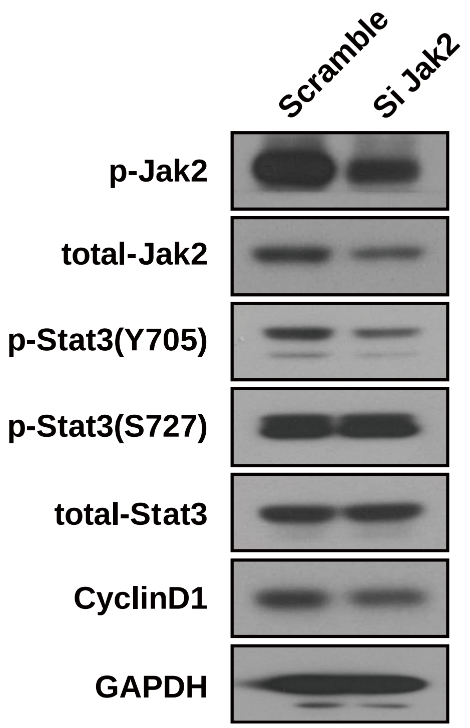

Supplementary Figure S5: Jak2 siRNA prohibits p-Stat3 (Y705) signaling pathway. PLC/PRF/5 cells were transfected with scrambled control or Jak2 siRNA for 48 h. Total cell lysates were prepared and western blots were carried out with antibodies of p-Jak2 (1007/1008), total Jak2, p-Stat3 (Y705), p-Stat3 (S727), total Stat3, and CyclinD1. GAPDH was used as a loading control. The results represent two independent experiments.

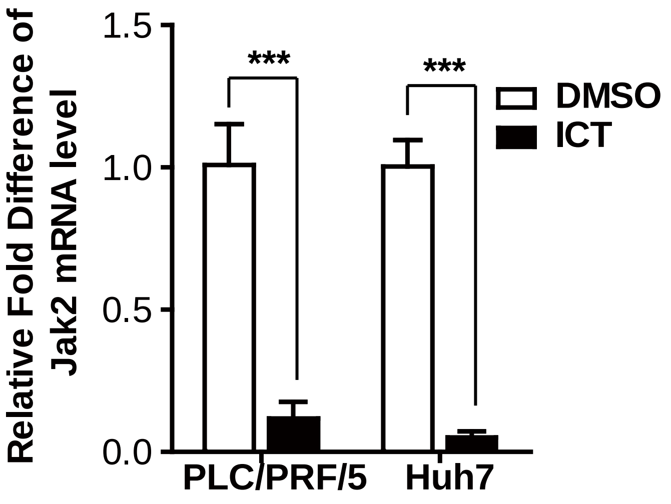

Supplementary Figure S6: Icaritin decreases the mRNA level of Jak2. PLC/PRF/5 and Huh7 cells were treated with 5 μM Icaritin for 24h. The mRNA level of Jak2 was evaluated by qRT-PCR using Jak2 primers. Columns, mean (n = 3); bars, SEM. ****P* <0.001.

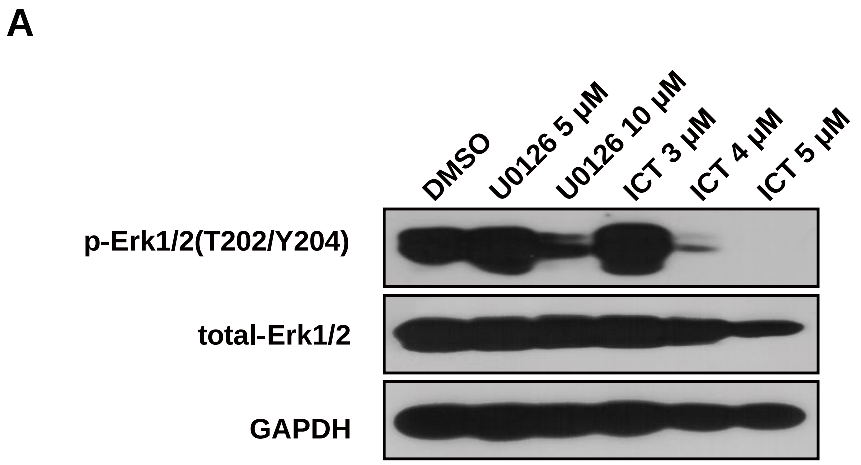

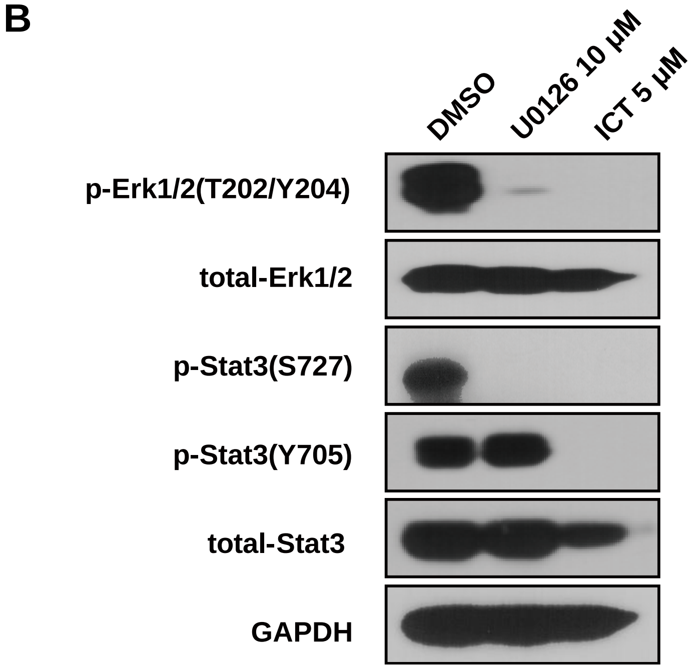

Supplementary Figure S7: Icaritin attenuates p-Stat3 (727) by blocking ERK1/2 pathway. (A) Huh7 cells were treated with DMSO or the indicated concentrations of U0126 or Icaritin for 48 h. Total cell lysates were prepared and western blots were performed using pERK1/2, total ERK1/2 and GAPDH antibodies. (B) Huh7 cells were treated with DMSO, 10 μM U0126, or 5 μM Icaritin respectively for 48 h. Total cell lysates were prepared and western blots were performed using pERK1/2, total ERK1/2, pStat3(Y705), pStat3(S727), total Stat3, and GAPDH antibodies. ICT, Icaritin. The results represent two independent experiments.

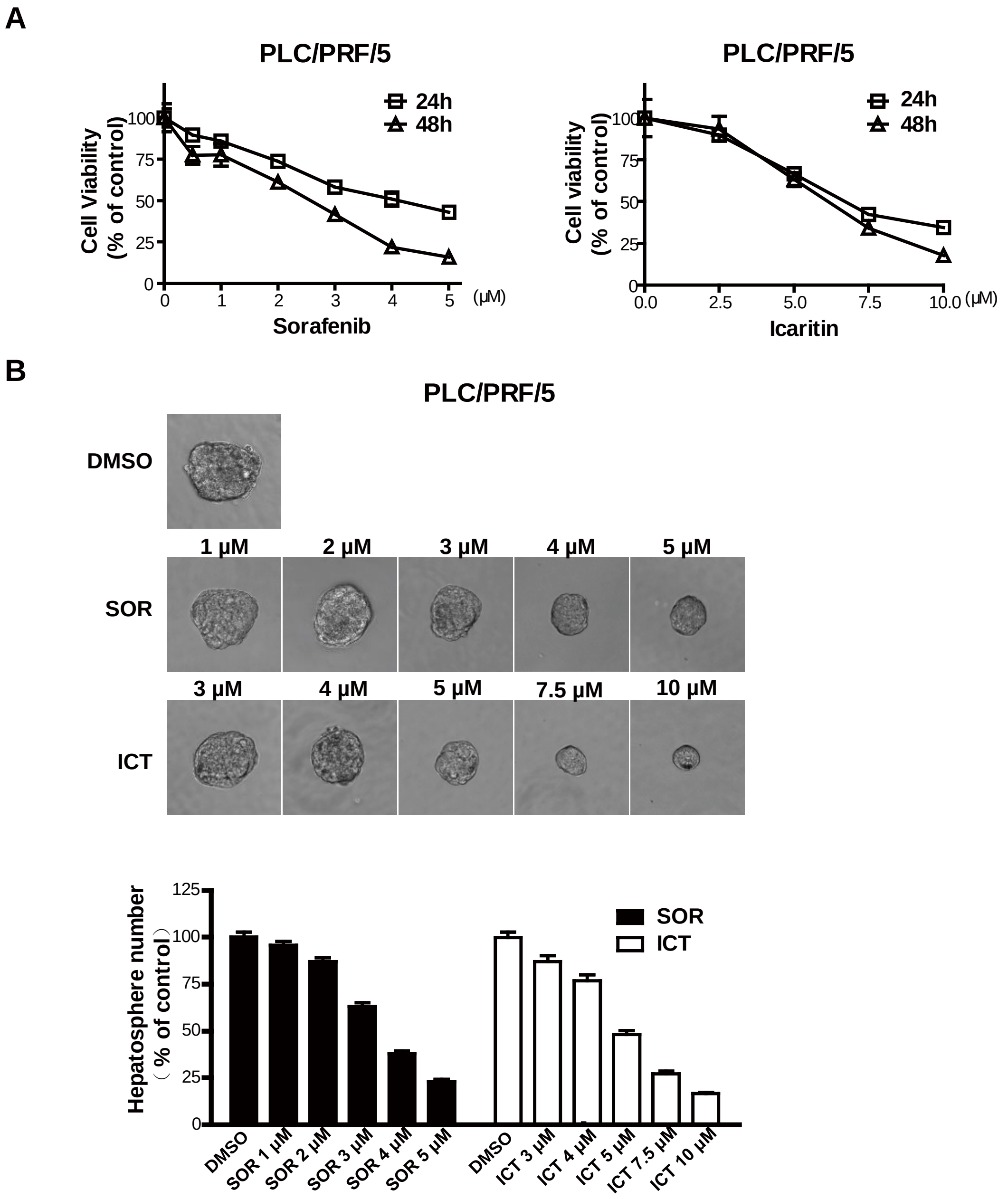

Supplementary Figure S8: Sorafenib and Icaritin suppress initiating cells of HCC. (A) PLC/PRF/5 cells maintained for 24 h in phenol red-free medium with 2.5% charcoal-stripped fetal calf serum were treated with Sorafenib (left) or Icaritin (right) at the indicated concentrations for 24 h or 48 h, and then cells were subjected to CCK8 assay. Each point represents mean (n=3) ± SD. (B) PLC/PRF/5 cells were pre-treated with the indicated agents for 48 h, then were seeded in low attachment sphere-forming medium. Five days later, hepatophere number was counted and analyzed. Columns, mean (n=3); bars, SEM; ICT, Icaritin; SOR, Sorafenib.

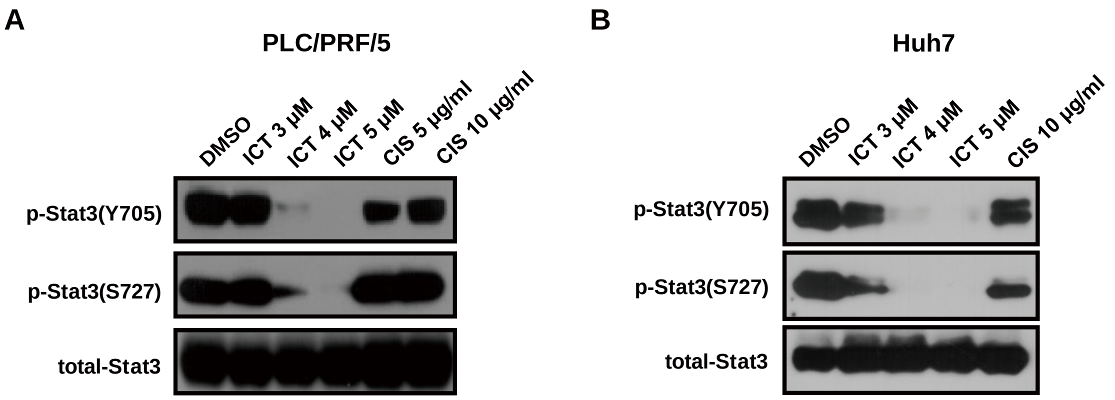

Supplementary Figure S9: Cisplatin has no inhibitory effect on phosphorylated Stat3. PLC/PRF/5 (A) and Huh7 (B) cells were treated with Icaritin or Cisplatin at the indicated concentrations for 24 h. Total cell lysates were prepared and western blots were performed using pStat3 (Y705), pStat3 (S727), total Stat3 antibodies. ICT, Icaritin; CIS, Cisplatin. The results represent two independent experiments.

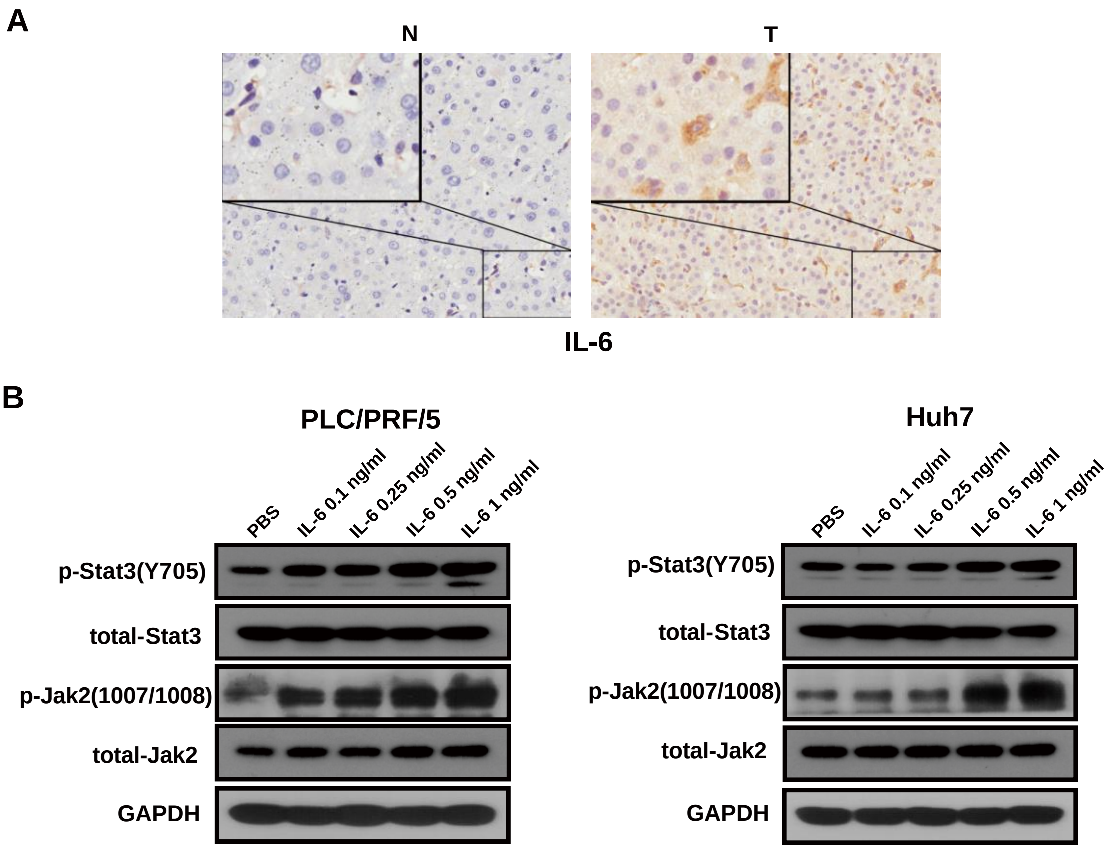

Supplementary Figure S10: IL-6 is highly expressed in HCC tumor tissues compared with normal liver tissue and induces phosphorylation of Stat3 and Jak2 in a dose-dependent manner. (A) IL-6 is highly expressed in HCC tumor tissues compared with normal liver tissue. Representative images of IL-6 IHC staining are shown. N: adjacent non-tumor tissue, T: HCC tissue (magnification: 200×). (B) IL-6 induces phosphorylation of Stat3 and JAK2 in a dose-dependent manner. PLC/PRF/5 and Huh7 cells were treated with PBS or the indicated concentrations of IL-6 for 1 h and cell lysates were prepared and western blots were carried out using antibodies of p-Jak2 (1007/1008), total Jak2, p-Stat3 (Y705), total Stat3 and GAPDH. The results represent two independent experiments.

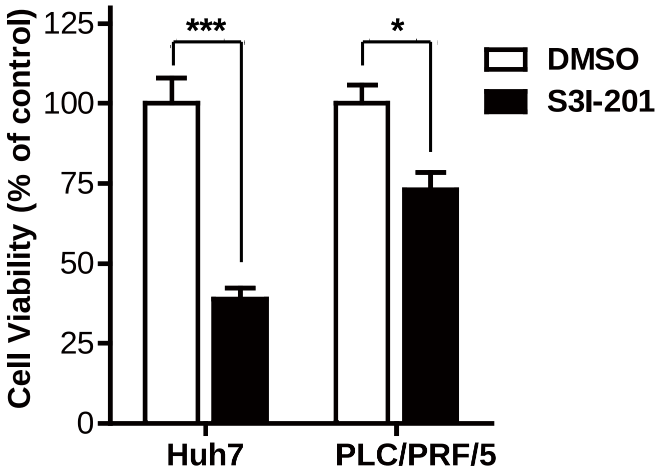

Supplementary Figure S11: The inhibitory effect of S3I-201 on cell viability. PLC/PRF/5 and Huh7 cells were treated with DMSO control or 150 μM S3I-201 (Stat3 inhibitor) for 48 h. After one day recovering, the living cells were selected with FACS and used for CCK8 assay in the absence of S3I-201. Columns, mean (n = 3); bars, SEM. *P<0.05; ***P < 0.001.

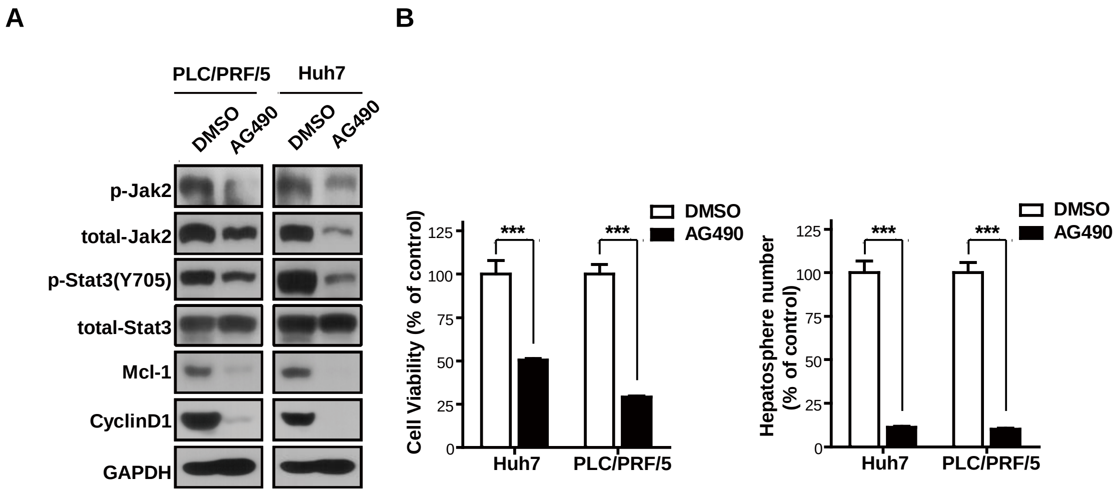

Supplementary Figure S12: Jak2 inhibitor prohibits HCC growth and initiation *in vitro*. (A) PLC/PRF/5 and Huh7 cells were incubated with DMSO control or 20 μM AG490 (Jak2 inhibitor) for 48 h. Cell lysates were harvested and western blots were carried out with antibodies of p-Jak2 (1007/1008), total Jak2, p-Stat3(Y705), total Stat3, Mcl-1, and CyclinD1. GAPDH was used as a loading control. The results represent two independent experiments. (B). PLC/PRF/5 and Huh7 cells were treated with DMSO control or 20 μM AG490 for 48 h. After one day recovering, the living cells were selected with FACS and carried out for the assays of CCK8 or hepatosphere counting in the absence of AG490. Columns, mean (n = 3); bars, SEM. ***, $P < 0.001$.

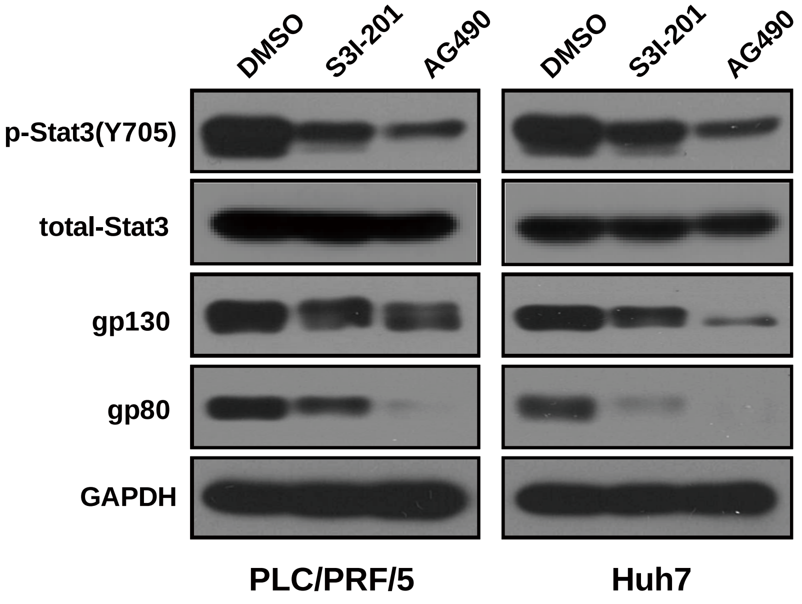

Supplementary Figure S13: S3I-201 and AG490 decrease the expression of gp130 and gp80. PLC/PRF/5 and Huh7 cells were treated with 150 µM S3I-201 or 20 µM AG490 for 24 h. Total cell lysates were prepared and western blots were performed using p-Stat3 (Y705), total Stat3, gp130, gp80 and GAPDH antibodies. The results represent two independent experiments.

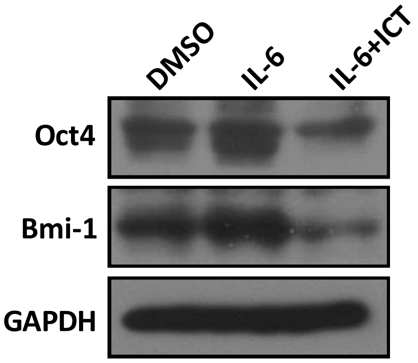

Supplementary Figure S14: Icaritin weakens IL-6-induced Oct4 and Bmi-1 expression. PLC/PRF/5 cells were incubated with IL-6 (20 ng/ml) in the absence or presence of 5 µM Icaritin for 24 hours. Total cell lysates were prepared and western blots were performed using Oct4, Bmi-1 and GAPDH antibodies. The results represent two independent experiments.

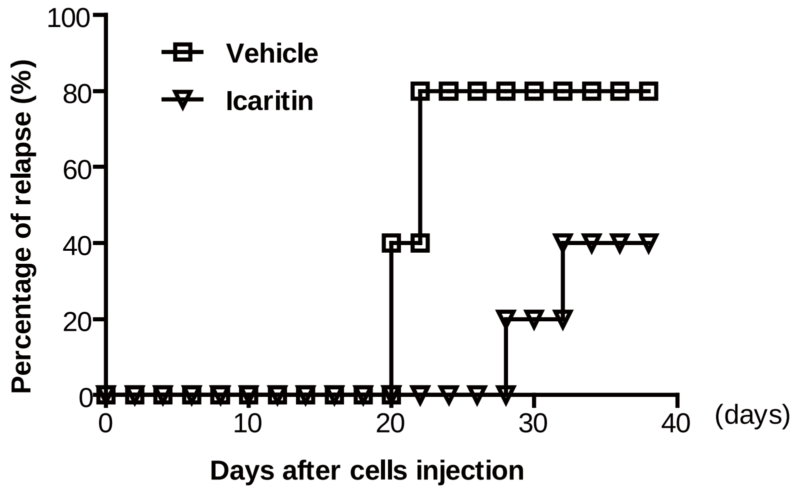

Supplementary Figure S15: Icaritin treatment retards PLC/PRF/5 secondary xenografts occurrence. Cells from each of vehicle-treated or Icaritin-treated PLC/PRF/5 xenografts were harvested and 1×10^4 living cells in each xenograft were used for secondary xenografts seeding. Tumor occurrence was evaluated as the percentage of relapse (n=5).

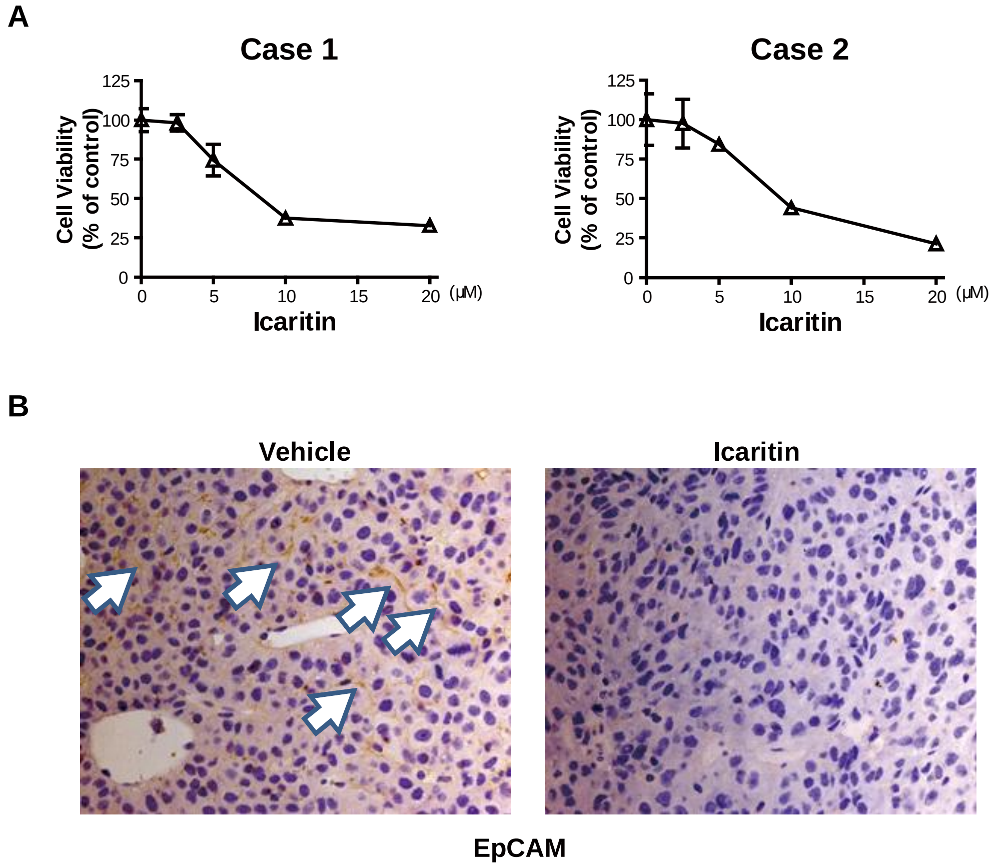

Supplementary Figure S16: Icaritin inhibits HCC patient derived cells growth *in vitro* and attenuates EpCAM$^+$ population in HCC patient derived xenografts. (A) Icaritin inhibits HCC patient derived cells growth. HCC patient derived cells (Case 1 and Case 2) were subjected with cell viability assay in the presence of DMSO (0) and 2.5, 5, 10, and 20 μM of Icaritin for 48 h. Data are expressed as percent of vehicle (DMSO) control. Each point represents mean (n=3) ± SD. (B) Icaritin treatment attenuates the EpCAM$^+$ population of Case 1-derived xenografts. Representative images of IHC staining of EpCAM are shown (magnification: 400×).

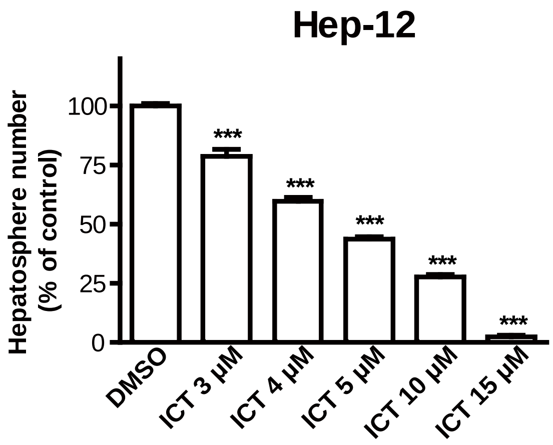

Supplementary Figure S17: Icaritin inhibits hepatosphere formation of Hep-12 patient derived cells. Hep-12 cells were cultured in low attachment sphere-forming medium in the presence of DMSO or the indicated concentrations of Icaritin. Hepatosphere number was counted 5 days later. Columns, mean (n = 3); bars, SEM. ***P <0.001.

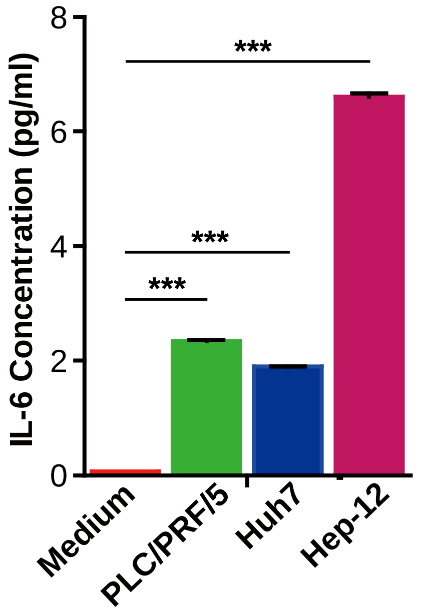

Supplementary Figure S18: HCC cells produce IL-6. The various HCC cells were cultured *in vitro* for 48 h, and then the supernatants of different cells or medium only control were collected. IL-6 concentrations were measured with ELISA assay according to the manufacturer's guidelines. Columns, mean (n=3); bars, SEM; ***P <0.001.

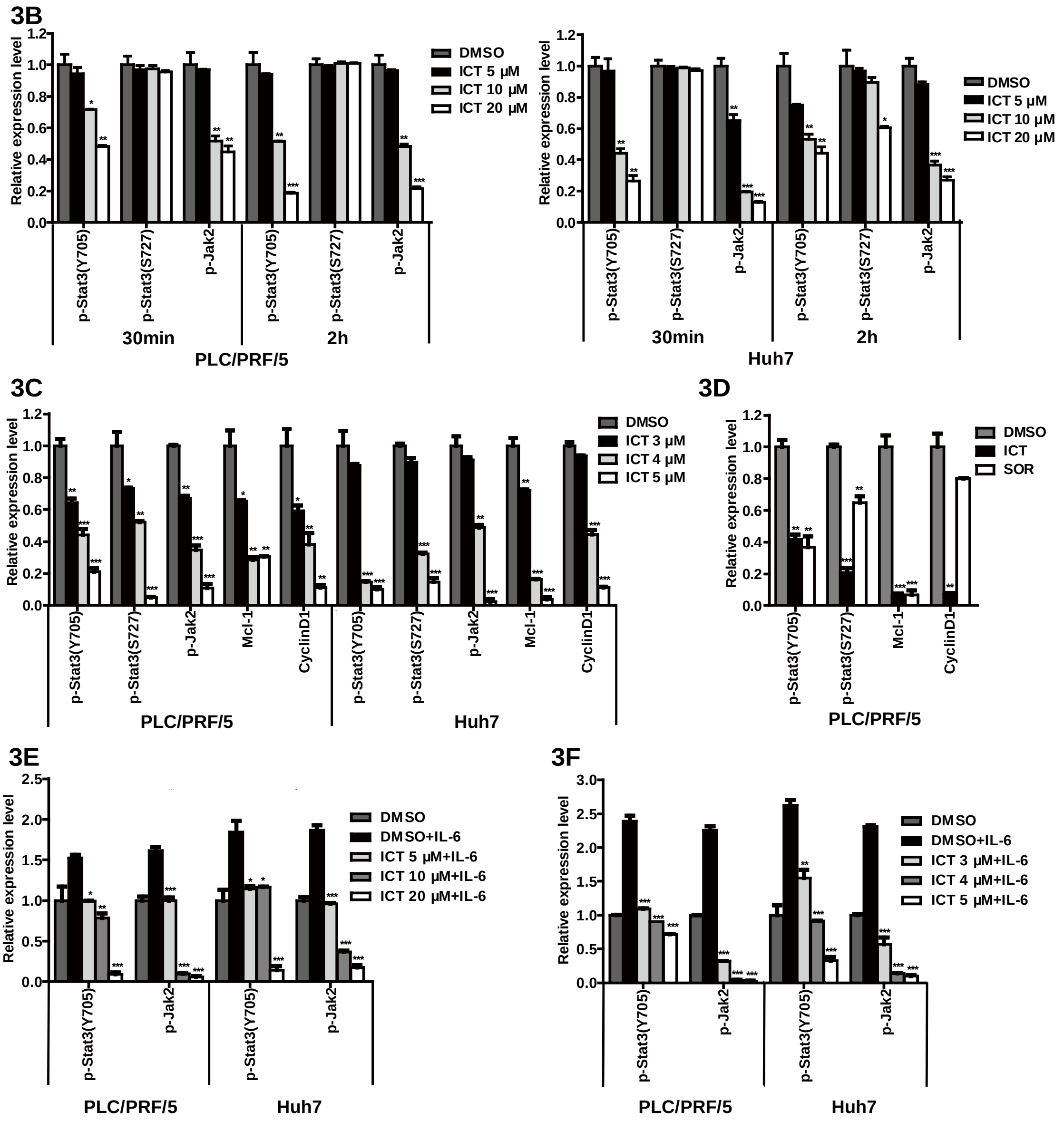

Supplementary Figure S19: Quantification Analysis of Western blots for Figure 3
*P<0.05; **P < 0.01;***P < 0.001.

4A

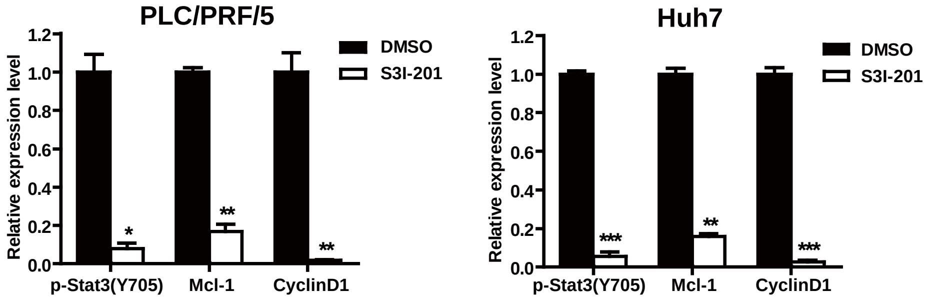

4C

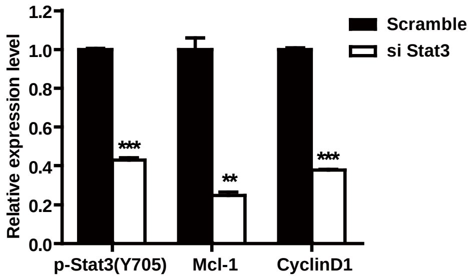

4E

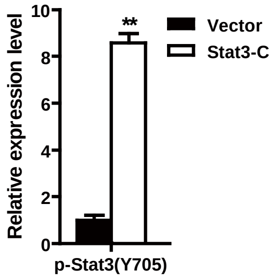

Supplementary Figure S19 continued: Quantification Analysis of Western blots for Figure 4 *$P<0.05$; **$P < 0.01$;***$P < 0.001$.

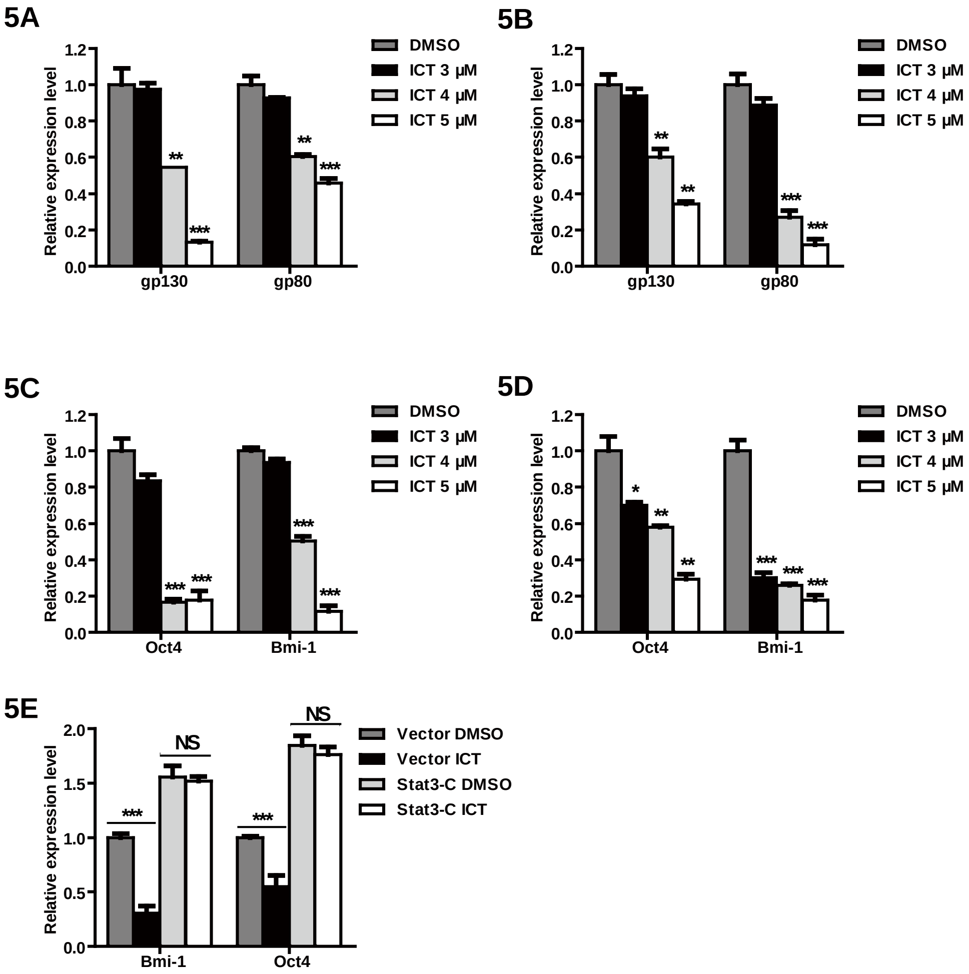

Supplementary Figure S19 continued: Quantification Analysis of Western blots for Figure 5 *P<0.05; **P < 0.01;***P < 0.001; NS: no statistic significance.

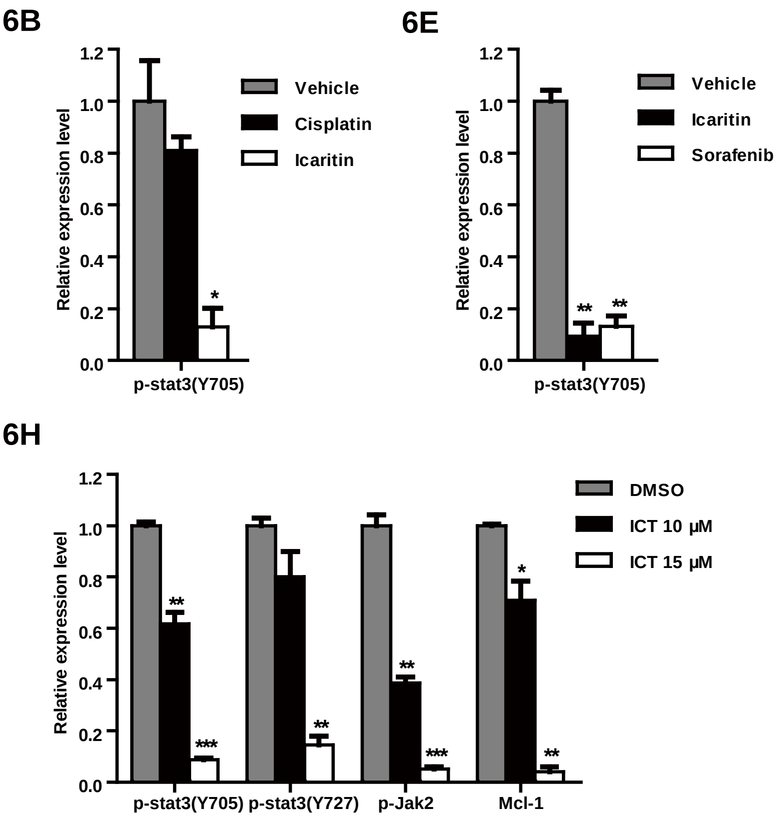

Supplementary Figure S19 continued: Quantification Analysis of Western blots for Figure 6 *P<0.05; **P < 0.01;***P < 0.001.

7D

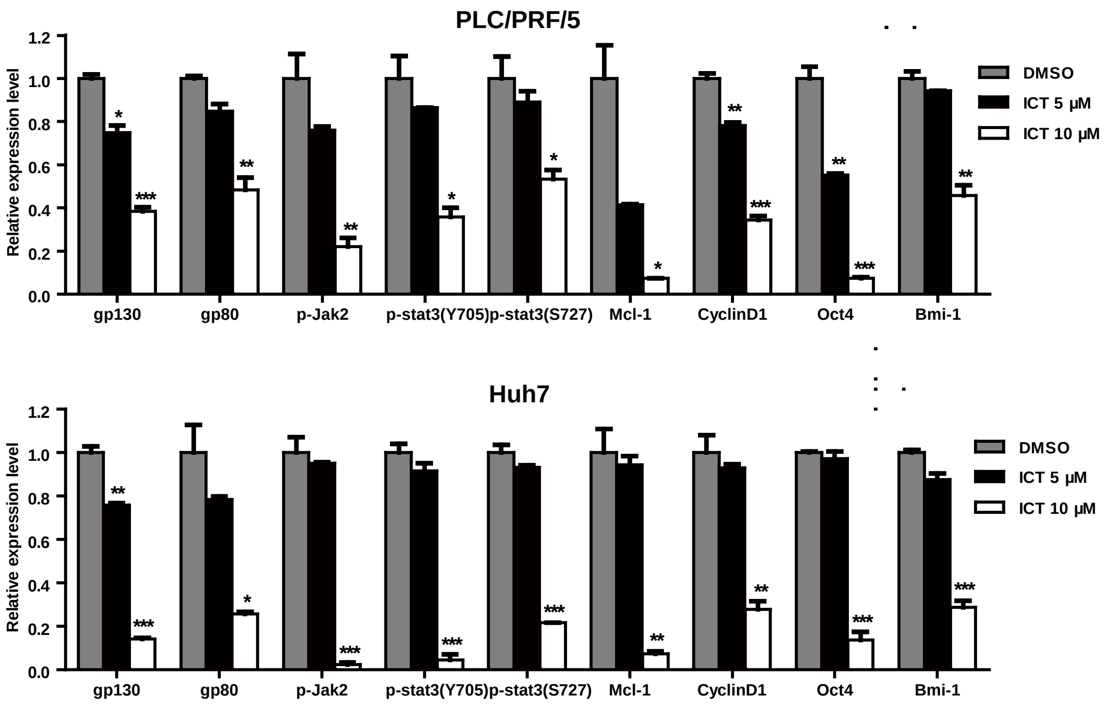

Supplementary Figure S19 continued: Quantification Analysis of Western blots for Figure 7 *P<0.05; **P < 0.01;***P < 0.001.

Supplementary Table S1

Clinicopathological characteristics of the patients

Characteristics	Case number	%
**Sex**		
Female	3	14.3
Male	18	85.7
**Age(years)**		
<50	6	28.6
>=50	15	71.4
**Hepatitis**		
HBsAg(+)	14	66.7
Anti-HCV(+)	2	9.5
No	5	23.8
**Cirrhosis**		
Yes	17	81.0
No	4	19.0
**AFP(ng/ml)**		
<=7	3	14.3
>7	18	85.7
**Tumor size(cm)**		
<=5	13	61.9
>5	8	38.1

**Vascular invasion**		
**Yes**	**2**	**9.5**
**No**	**19**	**90.5**
**Tumor differentiation**		
**Low**	**4**	**19.0**
**Moderate**	**14**	**66.7**
**High**	**3**	**14.3**

HBsAg, hepatitis B surface antigen; Anti-HCV, hepatitis C antibody; AFP, alpha-fetoprotein.

Supplementary Table S2

The list of primers name and sequence for qRT-PCR assay

Name	Forward primer(5’-3’)	Reverse primer(5’-3’)
EpCAM	GGGGAACAACTGGATCTGGA	CCAGCAACAACTGCTATCACC
CD133	AACGGCACCATTGGTCTCTG	AGGAAGGGAGGGAGTCATCC
CD24	TGAAGAACATGTGAGAGGTTTGAC	GAAAACTGAATCTCCATTCCACAA
Jak2	TTCAGCCAATGCAAAGCCAC	TCCTCACCTGAAGGACCACT
GAPDH	GAGTCAACGGATTTGGTCGT	GACAAGCTTCCCGTTCTCAG
